# eXtraembryonic ENdoderm (XEN) Stem Cells Produce Factors that Activate Heart Formation

**DOI:** 10.1371/journal.pone.0013446

**Published:** 2010-10-20

**Authors:** Kemar Brown, Michael Xavier Doss, Stephanie Legros, Jérôme Artus, Anna-Katerina Hadjantonakis, Ann C. Foley

**Affiliations:** 1 Greenberg Division of Cardiology, Weill Cornell Medical College, New York, New York, United States of America; 2 Developmental Biology Program, Sloan-Kettering Institute, New York, New York, United States of America; INSERM, France

## Abstract

**Background:**

Initial specification of cardiomyocytes in the mouse results from interactions between the extraembryonic anterior visceral endoderm (AVE) and the nascent mesoderm. However the mechanism by which AVE activates cardiogenesis is not well understood, and the identity of specific cardiogenic factors in the endoderm remains elusive. Most mammalian studies of the cardiogenic potential of the endoderm have relied on the use of cell lines that are similar to the heart-inducing AVE. These include the embryonal-carcinoma-derived cell lines, END2 and PYS2. The recent development of protocols to isolate eXtraembryonic ENdoderm (XEN) stem cells, representing the extraembryonic endoderm lineage, from blastocyst stage mouse embryos offers new tools for the genetic dissection of cardiogenesis.

**Methodology/Principal Findings:**

Here, we demonstrate that XEN cell-conditioned media (CM) enhances cardiogenesis during Embryoid Body (EB) differentiation of mouse embryonic stem (ES) cells in a manner comparable to PYS2-CM and END2-CM. Addition of CM from each of these three cell lines enhanced the percentage of EBs that formed beating areas, but ultimately, only XEN-CM and PYS2-CM increased the total number of cardiomyocytes that formed. Furthermore, our observations revealed that both contact-independent and contact-dependent factors are required to mediate the full cardiogenic potential of the endoderm. Finally, we used gene array comparison to identify factors in these cell lines that could mediate their cardiogenic potential.

**Conclusions/Significance:**

These studies represent the first step in the use of XEN cells as a molecular genetic tool to study cardiomyocyte differentiation. Not only are XEN cells functionally similar to the heart-inducing AVE, but also can be used for the genetic dissection of the cardiogenic potential of AVE, since they can be isolated from both wild type and mutant blastocysts. These studies further demonstrate the importance of both contact-dependent and contact-independent factors in cardiogenesis and identify potential heart-inducing proteins in the endoderm.

## Introduction

Studies in amphibians, avians and mice demonstrate that signals from both the dorsal midline [1], [2] and the endoderm [1], [3], [4], [5], [6], [7], [8], [9], [10] are essential for the initial specification of the cardiac mesoderm. However, experiments in which cells from the undifferentiated mouse epiblast were transplanted directly to the mesoderm (without traversing the primitive streak) demonstrated that midline signals are not strictly required for cells to adopt a cardiac fate [11]. This finding is consistent with our previous observations that signals from the dorsal lip of the frog activate heart formation indirectly by patterning the early endoderm [12], [13], [14]. Therefore, while signals from the dorsal midline are important for myocardial specification, it appears that the most proximate signal in this process comes from the endoderm.

In the mouse, both the AVE and AVE-like cell lines have been shown to direct nascent mesoderm toward cardiac fates [8], [15]. In addition, the AVE also plays an important role in patterning the anterior nervous system (reviewed in: [16], [17]). Mouse ES cells are pluripotent and, when grown as EBs, autonomously differentiate into cell types derived from all three of the three germ layers of the embryo, including rhythmically contracting cardiomyocytes [18], [19], [20], [21]. It is widely assumed that the ability of EBs to spontaneously form beating cardiomyocytes arises as a consequence of AVE formation within the EB, and indeed, endodermal signals enhance myocardial differentiation in both human and mouse EBs. Notably, the VE-like cell lines END2 [15], [22], [23], [24] and parietal endoderm-like PYS2 [25] mimic this effect. More recently, it has been shown that the AVE, and not the anterior definitive endoderm (ADE), induced by GATA4 overexpression in ES cells, possesses heart inducing ability [26].

Despite this growing body of work, the molecular nature of the heart-inducing signal in the AVE has yet to be identified. Moreover, it is not clear if the cardiogenic potential of the endoderm results from the activity of a secreted factor or through a contact-dependent effect of the endoderm. In addition, since both END2 and PYS2 cell lines were initially derived from embryonal carcinomas, it calls into question whether the cardiac-inducing signal(s) from these cells truly represent the endogenous heart-inducing signals in the embryo. The recent development of protocols to isolate XEN stem cells [27] represents a promising new approach that may overcome these difficulties. XEN cells are derived from blastocyst stage mouse embryos and represent the extraembryonic endoderm lineage: this being primitive endoderm in the blastocyst and parietal and visceral endoderm at early postimplantation stages. The visceral endoderm (VE) that overlies the anterior part of the epiblast is believed to be the source of cardiogenic inducing factors. We reasoned that since XEN cells are molecularly similar to the heart-inducing anterior AVE [27], [28], [29], they themselves might possess the ability to activate heart formation. In addition, since they can be derived directly from mouse embryos, the potential exists to isolate genetically modified XEN cells, for example from mutant strains of mice. In this way, mutant XEN cells can be used for a molecular dissection of the ability of extraembryonic endoderm to induce and pattern the heart.

Here, we compare the heart-inducing ability of XEN cells to that of END2 and PYS2 cells, whose heart inducing ability has been well-characterized (END2: [15], [22], [23], [24]; PYS2: [25]). We show that XEN cells, like END2 and PYS2 cells increase the likelihood that a region of beating cardiomyocytes will form within an EB and that both XEN and PYS2 cells increase the total number of cardiomyocytes that form during EB differentiation, suggesting that these cells not only activate the early steps of heart formation, but also produce factors involved in either the survival or proliferation of cardiomyocytes. In addition, all three cell lines increase cardiac marker expression, but with different temporal dynamics. Finally, comparative gene array analysis of these cell lines offers the first step in identifying factors that mediate specific steps of the cardiac specification program.

## Results

### XEN cells produce factors that increase myocardial differentiation

XEN cells, like END2 and PYS2 cells, express markers for the AVE [27], [28], [29], which has been proposed to be the heart-inducing endoderm tissue in the mouse embryo [8], [15]. Prompted by these observations, we sought to determine whether XEN cells can enhance myocardial differentiation from ES cells as has been shown for other extraembryonic endodermal cell lines.

**Figure 1:**
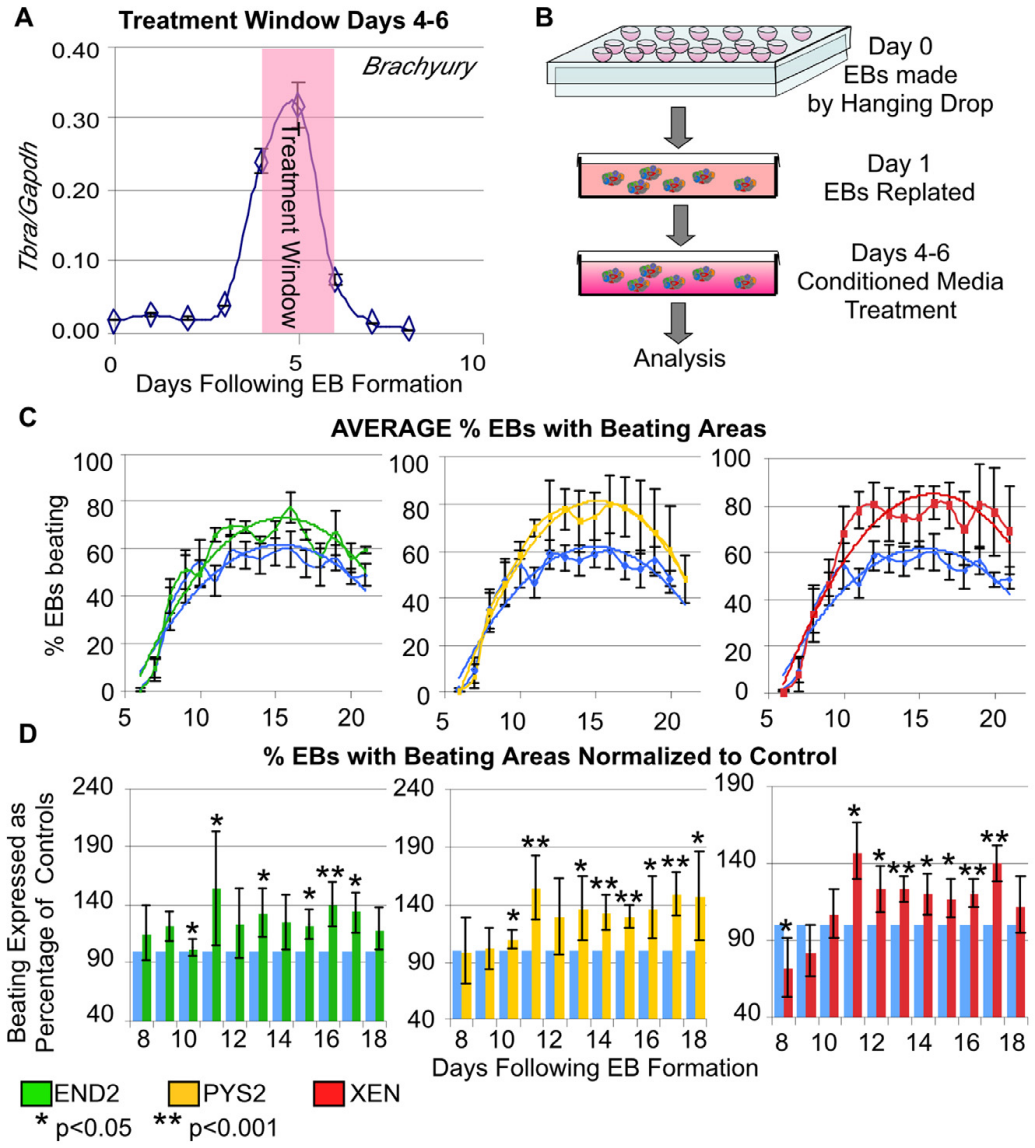
Addition of extraembryonic endoderm cell conditioned medium (CM) from day 4–6 of EB differentiation impacts whether EBs will form beating areas. Summary of data on the percentage of EBs that form beating areas in response to addition of media conditioned by END2, PYS2 and XEN cells. **A**. Treatment windows were determined by the expression of *Brachyury* in EBs as quantified by qRT-PCR. **B**. In these experiments, EBs were formed using a hanging drop method, and CM were added from days 4–6, during the peak of mesoderm formation (**A**). **C**. Data is represented as the average percentage of EBs within treated cultures that formed beating areas in response to addition of END2-CM (green), PYS2-CM (yellow) and XEN-CM (red) as compared to control cultures (blue). Polynomial trend lines are added to reveal the overall trend in these cultures. **D**. For a higher resolution view of the data, each experimental set (3–4 trials/condition) was normalized to controls (blue bars) for each day during the differentiation. Beating for each treated culture is displayed on each day as a percentage of beating in controls. (* indicates p-value<0.05, ** indicates a p-value<0.001).

Studies in avian and amphibian embryos suggest that endodermal signals are required only transiently during gastrulation to direct mesoderm towards heart formation [1]. Given this observation, we sought to investigate the temporal dynamics of mesoderm formation in EBs. As a marker of gastrulation we used *T/Brachyury*, which is expressed by the primitive streak and nascent mesoderm of the mouse embryo. We analyzed *Brachyury* expression in our cultures by qRT-PCR and determined that *Brachyury* expression is initiated approximately 3 days after EB formation, peaks at day 5, and is extinguished by day 7 (Figure 1A). We hypothesized, based on analogy to studies in other vertebrate organisms, that endodermal signals might preferentially exert an effect during the early phases of mesoderm formation. In addition, we were concerned that continuous addition of endodermally conditioned media might enhance myocardial differentiation by simply providing a nutrient rich environment that supports growth and survival. For this reason, in this assay, endodermally conditioned media was added to EBs only during this critical early phase of mesoderm formation (EB days 4–6) (Figure 1B) with treatment ending before the onset of beating.

Mouse embryonic stem (ES) cells possessing a transgene that drives green fluorescent protein (GFP) under the regulation of the *MHCα* promoter (*MHCα*::GFP) [30], a reporter of cardiomyocytes, were differentiated as EBs using standard serum-containing media. Under these conditions, expression of the *MHCα*::GFP reporter was first initiated at around day 6 of EB differentiation, the same time that cells within the EB began to beat rhythmically. All rhythmically contracting cells expressed the *MHCα*::GFP reporter (Movie S1). Conditioned media (CM) from END2, PYS2 and XEN cell lines were added, on days 4–6 (Figure 1A, B) (during peak mesoderm formation). From day 6 (first day of beating) until day 21, EBs were scored daily for beating and expression of the GFP reporter (Movie S2). Approximately 50 EBs were scored each day and beating was represented as a percentage of all EBs scored (Figure 1C: line graphs), demonstrating the overall trend in beating within the cultures. In control cultures, the percentage of EBs with beating areas peaks on day 16 with approximately 60% of EBs beating, whereas treatment with CMs increased this percentage to approximately 75%, 79% and 80% for END2, PYS2 and XEN-CM, respectively. To quantify this increase, beating was also normalized to beating in media controls. To do this the percentage of beating in treated cultures was represented on each day as a percentage of the beating found in the control cultures on the same day (Figure 1D: bar graphs). Addition of each of the three CMs resulted in a greater percentage of EBs that formed beating areas from day 10 onward.

**Figure 2:**
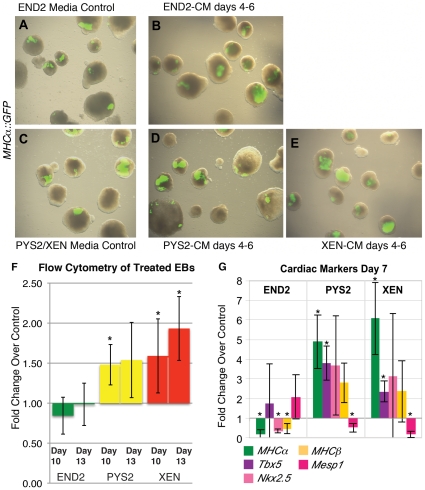
Addition of XEN-CM and PYS2-CM, but not END2-CM increases the amount of cardiomyocytes that form in culture and increases expression of cardiac markers as assessed by qRT-PCR. **A**, **C**. Merge of bright field and pseudo-colored fluorescence images to show distribution of cardiomyocytes in control EBs. A separate control is included for END2 cells since they are grown in different medium from the other two cell lines. **B**, **D**, **E**. Merge of bright field and pseudo-colored fluorescence images to show distribution of cardiomyocytes after treatment on days 4–6 with (**B**) END2, (**D**) PYS2 and (**E**) XEN-CM. **F**. Summary of flow cytometry data showing the fold change in the number of *MHCα*::GFP (+) cells on day 10 and 13 after addition of CM on days 4–6. (*indicates a p<0.05). **G**. qRT-PCR data showing expression of cardiac markers at day 7 after treatment of EBs with CM on days 4–6. (* indicates p<0.05).

We noted that while all three CMs increased the percentage of EBs that formed beating areas, beating areas remained relatively small in response to END2-CM. In contrast addition of PYS2-CM and XEN-CM increased both the number and size of the beating areas (Compare the difference Figure 2A and 2B to the differences between Figure 2C to 2 D, E). To further assess this effect, we performed flow cytometry on cells isolated from day 10 and day 13 EBs using the *MHCα*::GFP reporter to identify cells possessing a cardiac fate (Figure 2F). GFP-positive cells were analyzed and assessed as a percentage of total cells counted. Consistent with our data showing an increase in the number of EBs that beat, addition of XEN-CM or PYS2-CM from days 4–6 increased the percentage of GFP-positive cardiomyocytes on day 10. However by day 13, only EBs treated with XEN-CM continued to show a statistically significant increase in the number of cardiomyocytes. By contrast, treatment of EBs with END2-CM had no effect on the number of GFP-positive cells present at day 10 or day 13. Thus, while END2-CM increased the likelihood that an EB would form beating areas, it appeared that either cardiac progenitors did not expand after addition of END2-CM or that they failed to further supplement endogenous cardiogenic signals that arose from visceral endoderm formed by the EB. This finding was consistent with previous data demonstrating that CM from END2 cells increased cardiac differentiation of human ES cells [22], [23] but that co-culture was required to enhance cardiac differentiation from mouse ES cells [31].

To further assess the effect that these CMs have on cardiac progenitors, we investigated the expression of a panel of cardiac progenitor markers at day 7 (the second day at which beating was observed in our cultures and one day after termination of treatment). We determined the expression of both transcription factors (*Tbx5*, *Nkx2*.5 and *Mesp1*), which mark cardiac precursors, and cardiac contractile proteins (*MHCα*, and *MHCβ*). At day 7 of differentiation, EBs treated from days 4–6 with PYS2-CM or XEN-CM, but not those treated with END2-CM, exhibited increased expression of *MHCα*and the cardiac transcription factor *Tbx5* (Figure 2G). *Nkx2*.5 and *MHCβ* were also upregulated by PYS2-CM but with low statistical probability (p-value = 0.21 and 0.08 respectively). *Mesp1* [*32*] was downregulated in response to XEN-CM and PYS2-CM. After addition of END2-CM, cardiac markers were either downregulated or unaffected on day 7. This suggests that PYS2-CM and XEN-CM, but not END2-CM, expand the size of the cardiac progenitor pool present in EBs.

**Figure 3:**
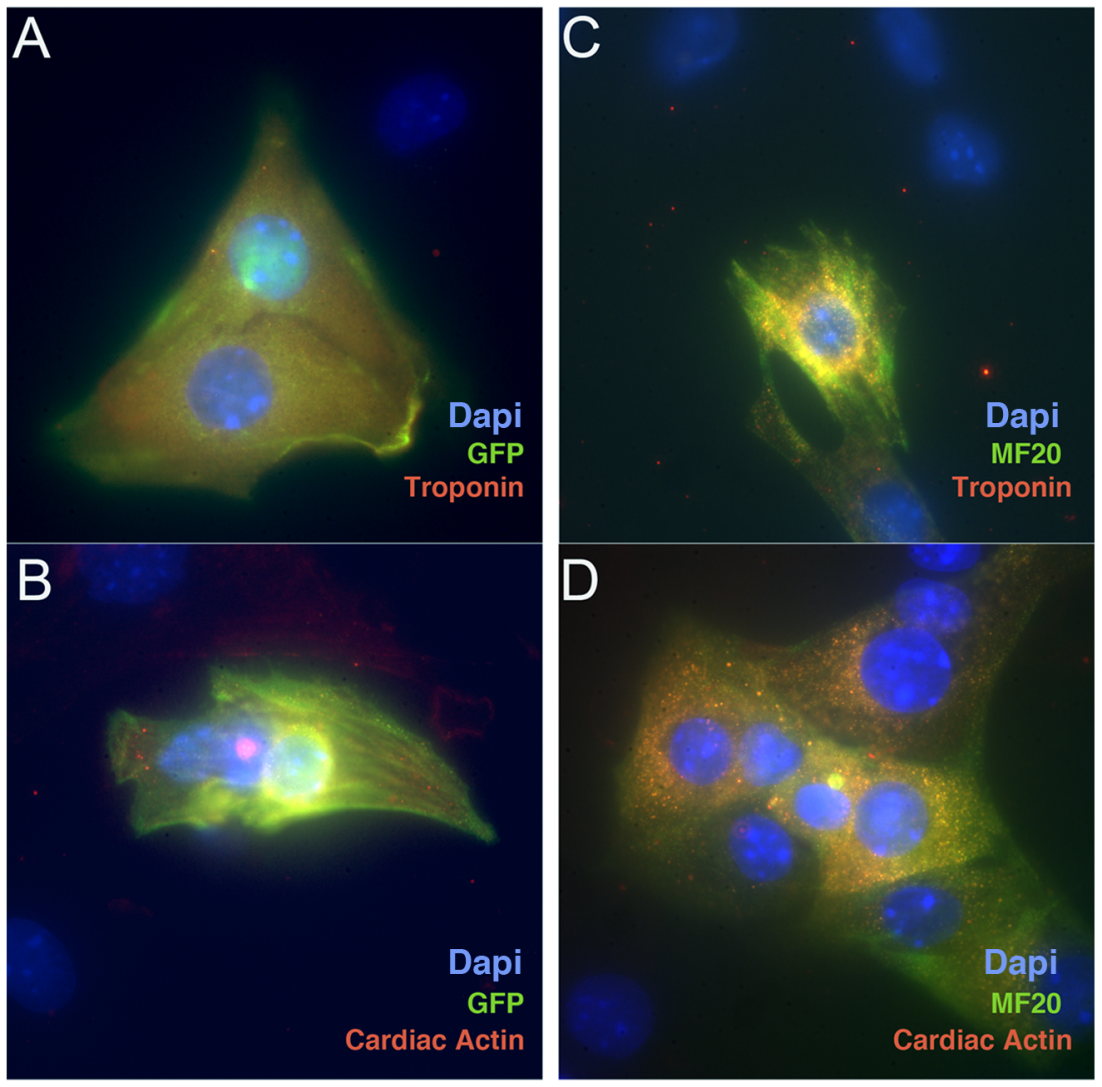
Immunocytochemistry of MHCα::GFP(+) cells induced by XEN-CM. EBs treated from days 4–6 with XEN-CM were dissociated as described for flow cytometry analysis. They were then fixed and processed for immunoctyochemistry using antibodies that recognize either GFP (green in **A** and **B**) or the cardiac specific epitopes Troponin (red in **A** and **C**), Cardiac Actin (red in **B** and **D**) and cardiac-specific Myosin (MF20) (green in **C** and **D**). These studies demonstrate the correlation between cardiac markers and GFP.

Our flow cytometry analysis of these cells was based on the expression of GFP driven by the MHCα promoter. This cell line has been previously described [30]; however, we wanted to confirm that the GFP reporter faithfully reflected cardiomyocyte differentiation and wanted to show that treatment with XEN-CM neither activates a non-cardiac cell type expressing the *MHCα*::GFP reporter nor increases the stability of GFP in cells that may have previously expressed cardiac markers transiently, but no longer do so. To address these possibilities, EBs that had been treated with XEN-CM were dissociated using the same protocol as was used for the flow cytometry studies, and plated onto gelatin-coated chamber slides. 24 hours after plating, cells were observed by fluorescence microscopy. At this point, GFP–positive cells were seen as rhythmically contracting cells in these dissociated cultures. By contrast, no contractions were observed in GFP-negative cells. Cells were then fixed and processed by immunocytochemistry for antibodies recognizing GFP and the cardiac-specific epitopes, Troponin, Cardiac Actin and Cardiac-specific Myosin (MF20). We noted that GFP-positive cells also expressed Troponin and Cardiac Actin (Figure 3A, B). There was also coexpression between these cardiac markers and cells recognized by the MF20 antibody, which recognizes cardiac-specific myosin (Figure 3C, D). These findings demonstrate that *MHCα*::GFP is a faithful reporter of cardiac fates and the addition of XEN-CM does not activate non-cardiac GFP or enhance the perdurance of GFP.

**Figure 4:**
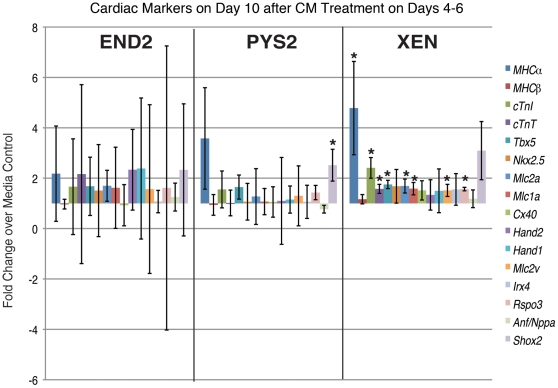
Cardiac Marker expression on Day 10 following treatment on days 4–6. qRT-PCR data showing expression of a panel of general and region-specific cardiac markers at day 10 after treatment of EBs with CM on days 4–6. (* indicates p<0.05).

We also determined the expression of these markers at day 10 (Figure 4) and noted that only EBs treated with XEN-CM continued to show statistically significant increases in the expression of cardiac markers including, *MHCα*, *Tbx5*, *cTnI*, *cTnT*, *Tbx5*, *Mlc2a and Mlc1a*, *Mlc2v*. While the expression of regional-specific markers has not been rigorously assessed during EB differentiation, we noted that most of the markers upregulated show biased expression toward the atria or inflow tract of the embryonic heart (MHCα, Mlc1a, Mlc2a, cTnI [33], Tbx5 [34], [35]), whereas markers that are more strongly expressed in the ventricles (Hand1 [36], [37], Hand2 [37] and Irx4 [38]) were not significantly altered as compared to controls. Expression of the conduction system marker *Connexin-40* (Cx40) [39], and the secondary heart field marker, *Islet1* [40] (data not shown) were also not significantly different between treated EBs and controls. Taken together these data suggest that factors in XEN-CM may bias cardiomyocyte differentiation toward an atrial fate. To further address this, we analyzed the expression of atrial specific markers *ANF/Nppa* and *Shox2*, which mark the whole atrium and the right atrial wall, respectively. *Nppa* was not significantly different between treated and untreated EBs (not shown) and only PYS2-CM treated EBs showed a statistically significant increase in *Shox2* expression, which marks the sinus venosus, and later, the dorsal wall of the right atrium [41]. Together, these findings suggest that endodermal CMs do not affect the overall patterning of the myocardium.

Finally, since addition of END2-CM only enhanced beating in our cultures after day 13, we also assessed *MHCα* expression on day 16. We found that even at this stage, *MHCα* expression was enhanced by PYS2-CM and XEN-CM, but not by END2-CM (data not shown). These findings demonstrate that although addition of END2-CM affects the likelihood that an EB will form rhythmically contracting cardiomyocytes, END2-CM either lacks signals supporting the subsequent proliferation of cardiomyocytes or causes a non-specific increase in proliferation of all cell types within the EB, thereby masking any effect that it might have on cardiomyocyte differentiation. To address this, we measured the overall growth rate of EBs during and after treatment with conditioned media by counting the total number of cells in 20 EBs on each day of culture. We found no difference in the growth rate of EBs treated with conditioned medium (data not shown). This suggests that endodermally conditioned media may specifically affect the proliferation or survival of differentiating cardiomyocytes in EBs, but not the overall rate of cell division.

### The cardiogenic activity of endodermal cells is sensitive to timing

**Figure 5:**
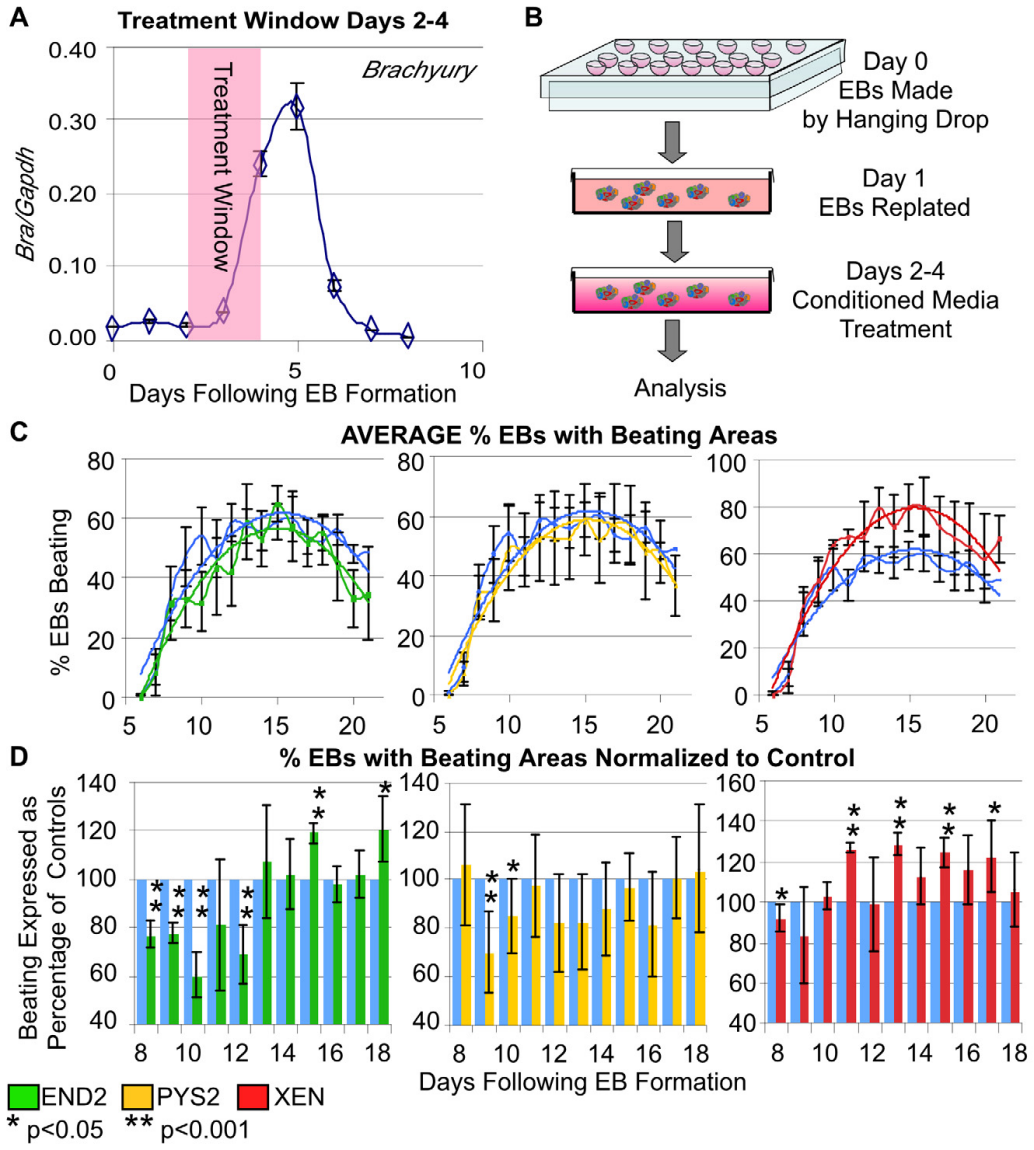
Addition of extraembryonic endodermal conditioned medium (CM) from days 2–4 of EB differentiation either had no impact on beating or delayed the formation of myocardial cells. Summary of data showing the effect of conditioned media on the percentage of EBs that form beating areas in response to addition of media conditioned by END2, PYS2 and XEN cells. **A**. Treatment windows were determined by the expression of *Brachyury* in EBs as determined by qRT-PCR. **B**. In these experiments EBs were formed using a hanging drop method, and CM were added from days 2–4, prior to the onset of mesoderm formation (A). **C**. Data is represented as a percentage of EBs within treated cultures that formed beating areas in response to addition of END2-CM (green), PYS2-CM (yellow) and XEN-CM (red) as compared to control cultures (blue). Polynomial trend lines are added to reveal the overall trend in these cultures. **D**. For a higher resolution view of the data, each experimental set (3–4 trials/condition) was normalized to controls (blue bars) for each day during the differentiation and beating is displayed on each day as a percentage of beating in the control condition on that day. (* indicates p-value<0.05, ** indicates a p-value<0.001).

**Figure 6:**
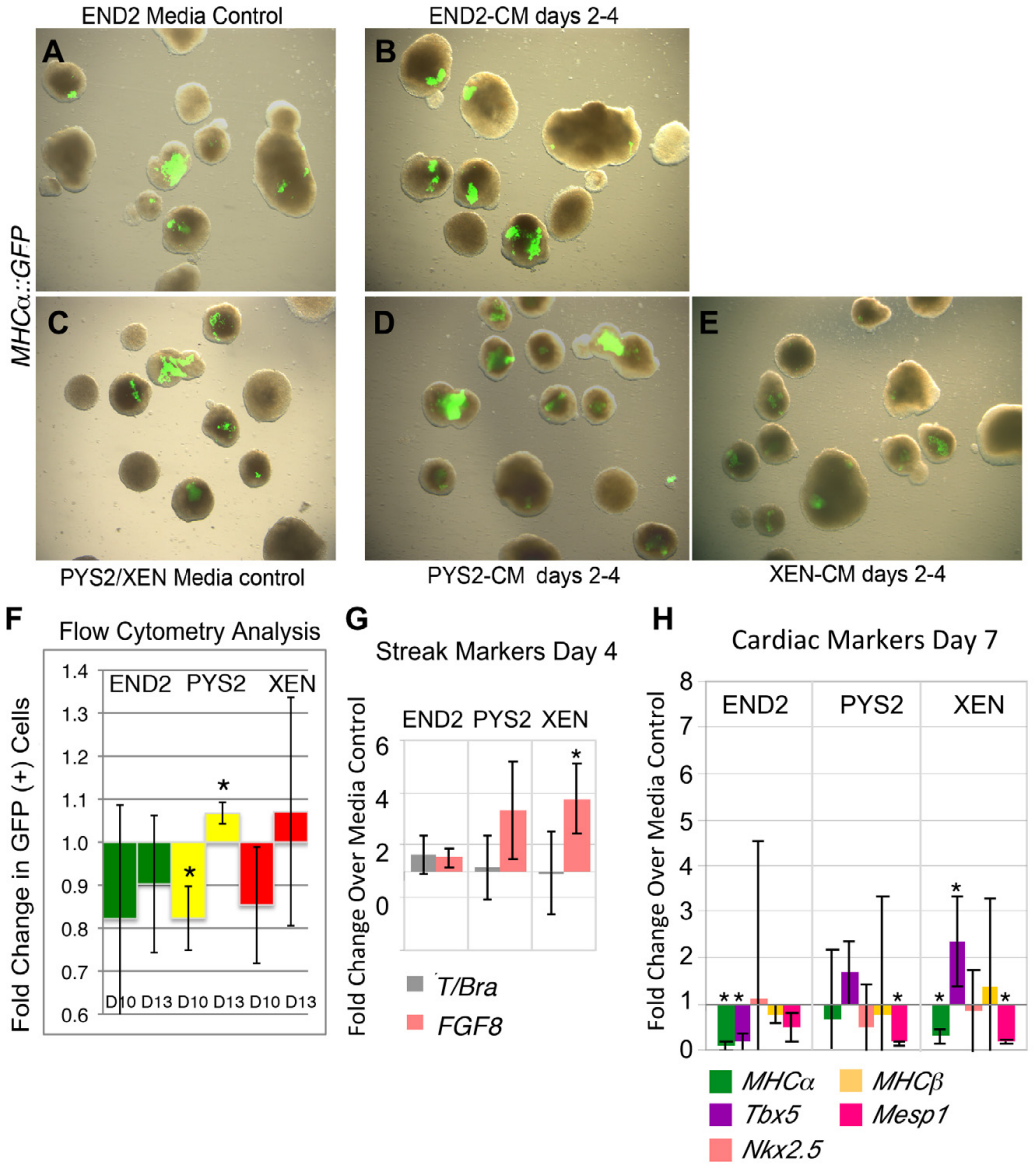
Effect of CM on cardiomyocyte differentiation when added before mesoderm formation. **A**, **C**. Merge of bright field and pseudo-colored fluorescence images to show distribution of cardiomyocytes in control EBs. A separate control is included for END2 cells since they are grown in different medium from the other cell lines. **B**, **D**, **E**. Merge of bright field and pseudo-colored fluorescence images to show distribution of cardiomyocytes after treatment on days 2–4 with (**B**) END2, (**D**) PYS2 and (**E**) XEN-CM. **F**. Summary of flow cytometry data showing the fold change in the number of *MHCα*::GFP (+) cells on days 10 and 13 after addition of CM on days 2–4. (*indicates a p<0.05). **G**, **H**. qRT-PCR data showing expression of mesoderm markers at days 4 and cardiac markers on day 7 after treatment of EBs with CM on days 2–4. (* indicates p<0.05).

Studies of cardiac specification in various animal models reveal a strict temporal requirement for signals from the endoderm involved in this process [1], [42]. To test this, EBs were treated with CM in an earlier time window, between days 2–4, which corresponds to a period just prior to mesoderm formation in our cultures (Figure 5A, B). One possible mechanism by which endodermal signals might enhance myocardial differentiation is by increasing mesoderm formation. If this were the case then it might be expected that addition of CMs at this earlier time point might further increase cardiac differentiation. As before, we tested whether addition of CM could increase the percentage of EBs that formed beating areas. EBs were assessed from day 6 (first day of beating) until day 21, for beating and activity of the *MHCα*::*GFP* reporter. Approximately 50 EBs were scored each day, and beating was represented as a percentage of all EBs scored (Figure 5C: line graphs) to show the overall trend in beating within the cultures. Beating was also normalized to controls and represented on each day as a percentage of beating in controls on that day (Figure 5D: bar graphs). In contrast to the results obtained when CM was added during the peak of *Brachyury* expression, when EBs were treated from day 2–4 (prior to mesoderm formation) CM either had no effect on, or delayed the onset of beating (Figure 5D). Only XEN-CM enhanced beating when added in this time window. To confirm this, we performed flow cytometry on cells isolated from day 10 EBs using *MHCα*::GFP reporter for cells possessing a cardiac fate (Figure 6A–E). GFP-positive cells were analyzed and assessed as a percentage of total cells counted. Addition of endodermal-CM from days 2–4 had either no impact or a negative impact on the percentage of GFP-positive cells at day 10 as compared to controls (Figure 6F). This was consistent with EB counting data on day 10. These cultures were also analyzed by flow cytometry at day 13. There was a small but statistically significant increase after treatment with PYS2-CM, but otherwise there were no differences between treated and untreated EBs at these time points (Figure 6F). As before, we also assessed myocardial specification in response to endodermal CM by using qRT-PCR to assess for changes in the expression of mesoderm markers generally and cardiac markers specifically. At day 4, (just after the termination of CM treatment) we examined the expression of markers for the nascent mesoderm (Figure 6G). None of the CMs increased expression of *Brachyury*, but addition of XEN-CM in this time window did result in a statistically significant increase of *Fgf8* expression.

To assess the effect that this earlier treatment had on the expression of cardiac progenitor markers, we examined the expression of the cardiac contractile proteins *MHCα* and *MHCβ*, as well as the transcription factors *Nkx2.5*, *Tbx5* and *Mesp1* (Figure 6H) on day 7 of differentiation. EBs treated with END2-CM exhibited decreased expression of *MHCα* and *Tbx5* as compared to controls. Other cardiac markers were unaffected in response to END2-CM. By comparison, PYS2-CM and XEN-CM resulted in a decrease in expression of *Mesp1*. In general, treatment in this time window resulted in an overall decrease in the expression of cardiac progenitor markers.

**Figure 7:**
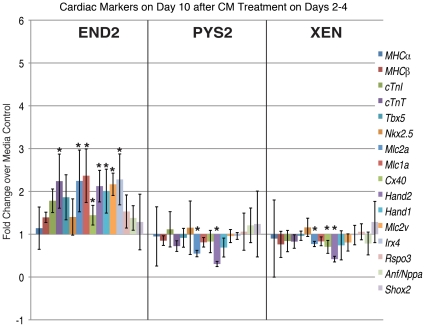
Cardiac marker expression on day 10 following treatment on days 2–4. qRT-PCR data showing expression of a panel of general and regional specific cardiac markers at day 10 after treatment of EBs with CM on days 2–4. (* indicates p<0.05).

To test the effect of endodermally conditioned media on overall cardiac differentiation, we also assessed a panel of cardiac markers on day 10 of differentiation (Figure 7). Treatment of PYS2-CM or XEN-CM on days 2-4 resulted in a downregulation of *Mlc2a* and *Hand2* at day 10, whereas other markers were not significantly different from controls. Interestingly, EBs treated with END2-CM on days 2–4 exhibited a general upregulation of cardiac marker expression on day 10. This data does not correlate with either an overall increase in the number of cardiomyocytes or an increase in the percentage of EBs with beating areas. Indeed day 10 represented a low point in terms of percent beating as compared to controls (Figure 5D), This finding tells us that mRNA expression does not necessary correlate temporally with cardiac fate. Therefore cardiac induction can only be assessed by a comparison of molecular, functional and marker analysis at several time points, such as we present here.

### Microarray analysis

**Figure 8:**
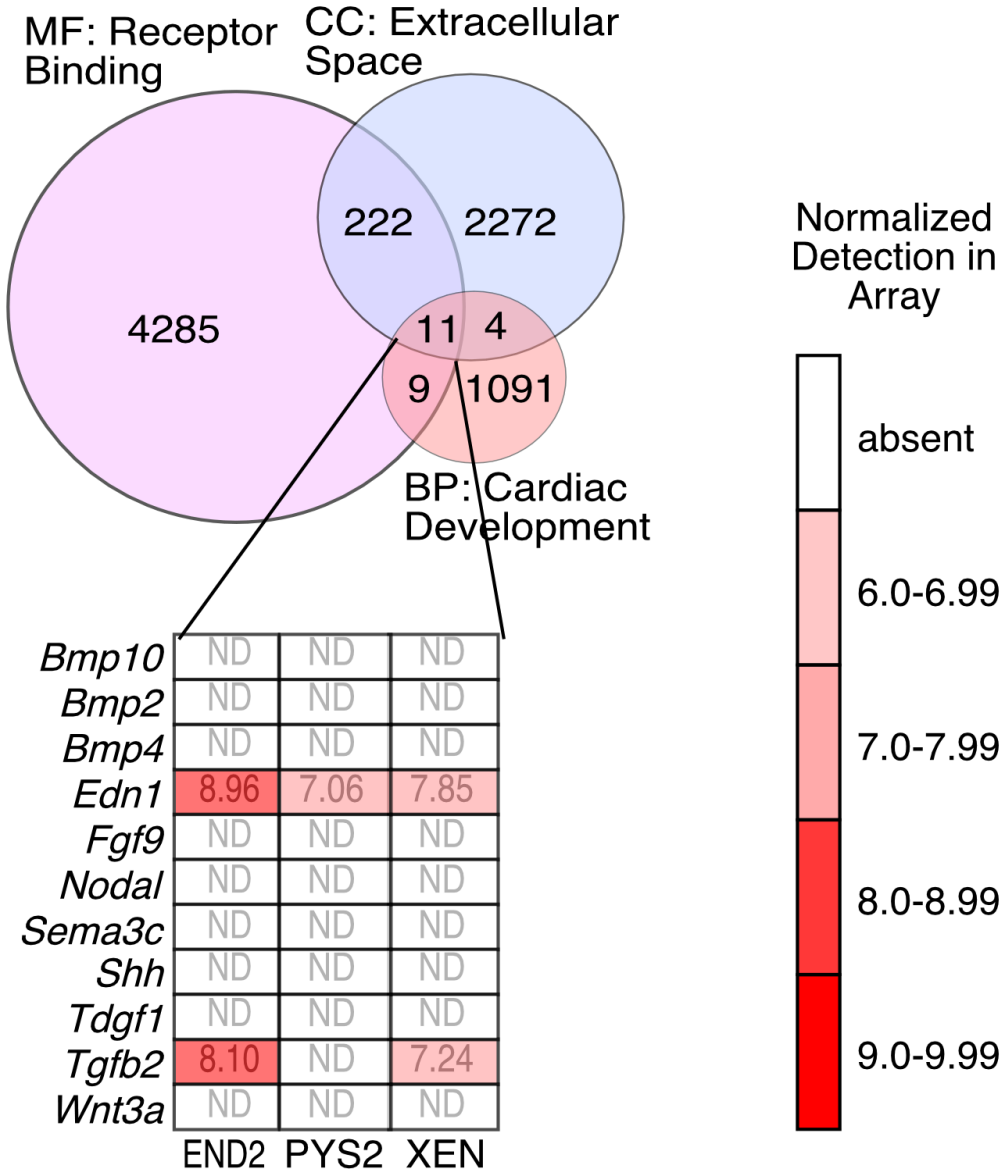
Growth factors secreted by heart inducing cell lines. Venn diagram showing overlapping transcripts among the Gene Ontology (GO) Consortium classifications BP: cardiac development (GO term: 0007507), MF: receptor binding (GO term: 0005102) and CC: extracellular space (GO term: 0005615). Eleven factors were found to be present in all three data sets and are represented as a heat map. Of these, only two factors, *Endothelin* and *TGFbeta2* were detected in the arrays (based on a p-value of detection less than 0.01). The data discussed in this publication have been deposited in NCBI's Gene Expression Omnibus [73] and are accessible through GEO Series accession number GSE19564 (http://www.ncbi.nlm.nih.gov/geo/query/acc.cgi?acc=GSE1956.

Our analyses not only demonstrate that XEN cells have cardiac inducing ability but this also reveal differences between XEN cells and other heart-inducing endodermal cell lines. We have previously performed an extensive comparison of microarray data from END2, PYS2 and XEN cells [28]. In this previous study, we validated these arrays by demonstrating a high (greater than 80%) correlation between array data and our other methods (for example qPCR) of quantifying gene expression within these cells. Prompted by our finding that XEN cells, like previously characterized heart-inducing cells, have the ability to activate heart formation, we re-analyzed these arrays. Specifically, we sought [28] to identify secreted factors that might account for the contact-independent ability of endodermal cell lines to support myocardial differentiation. To accomplish this, we compared gene rosters for ontology terms classified by the Gene Ontology (GO) Consortium (http://www.geneontology.org) [43] as “cardiac development”, “extracellular space”, and “receptor binding”, and identified a list of 11 common genes (Figure 8). Of these genes only two, *Endothelin1* and *Tgfbeta2*, were detected in the arrays, and of these only *Endothelin1* is expressed by all three heart-inducing extraembryonic endoderm cell lines. This finding suggests that the cardiogenic factors secreted by endodermal cell lines have yet to be characterized and classified by GO.

**Figure 9:**
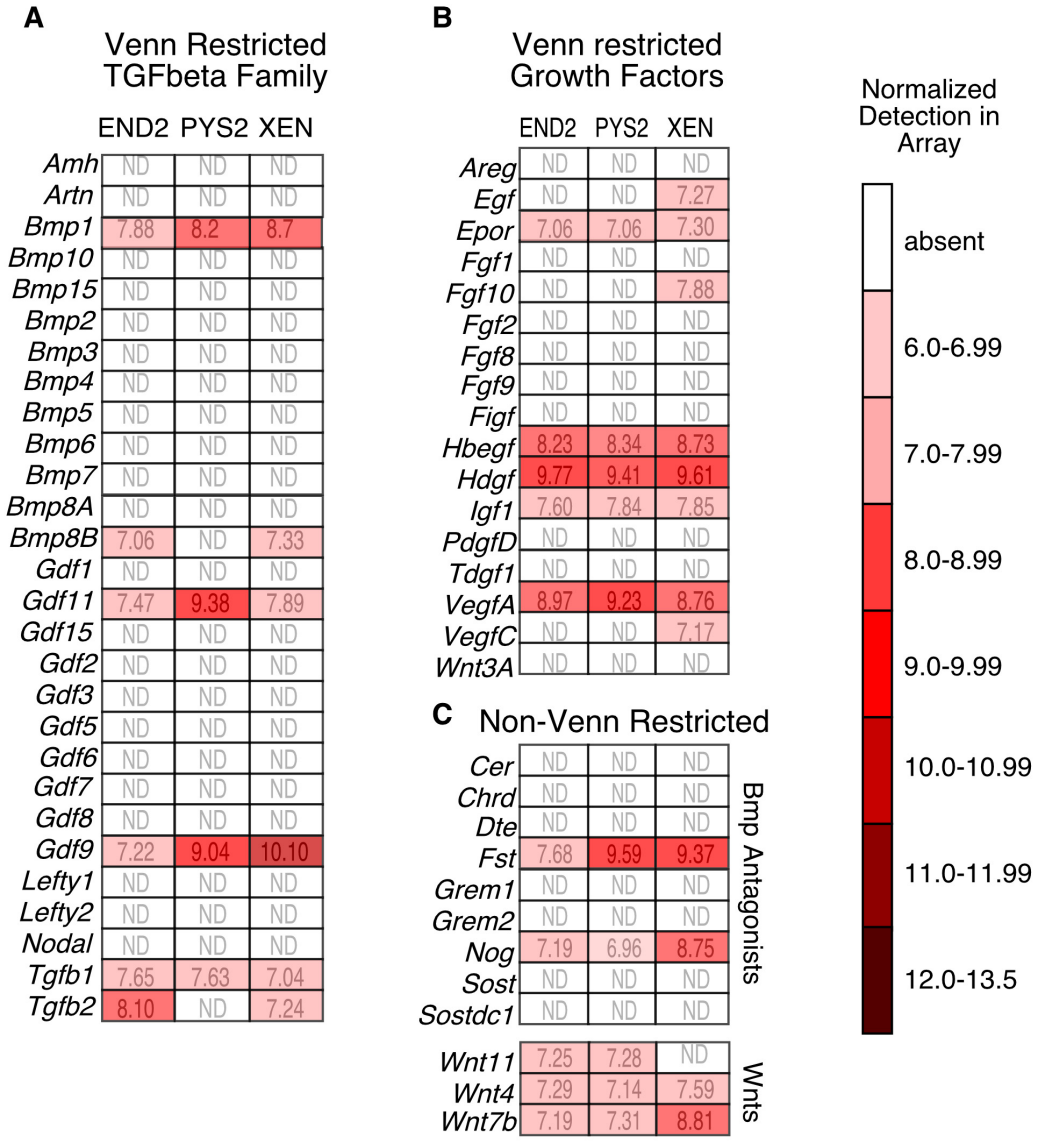
Venn-restricted factors expressed by heart inducing cell lines. **A**. Expression of Venn-restricted TGFbeta family members in the subset defined by GO terms [receptor binding] and [extracellular space]. Normalized array data are expressed as a heat map. **B**. Expression of Venn-restricted growth factors in the array in the subset defined by GO terms [receptor binding] and [extracellular space]. **C**. Expression of BMP antagonists and Wnt family members (not Venn-restricted) expressed in the array. Probes are called as present if the p-value for detection was less than 0.01.

**Figure 10:**
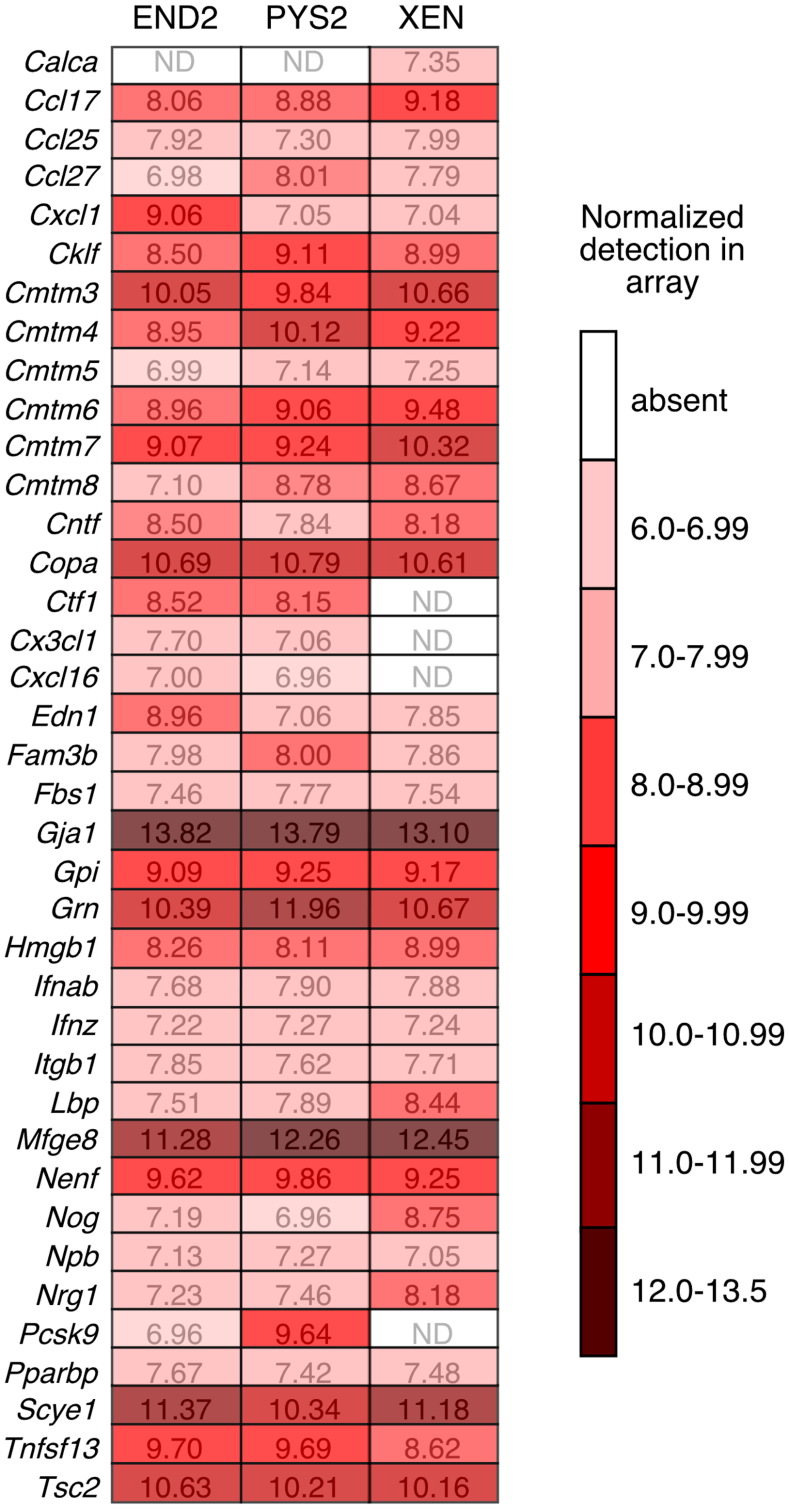
Additional extracellular factors identified by Gene Ontology are expressed by heart inducing cell lines. Normalized expression in the array, expressed as a heat map, of the remaining 38 Venn-restricted factors that were detected as present in the array based on a detection p-value<0.01.

To expand the list of candidate secreted factors that might account for the cardiac inducing ability of these extraembryonic endodermal cell lines, we analyzed the expression of the remaining 222 Venn-restricted genes. The largest group (comprising 28 genes) of the remaining Venn-restricted genes were members of the TGFbeta superfamily of signaling molecules (Figure 9A). Of these, the three extraembryonic endodermal cell lines expressed only six genes, including *TGFbeta-1* and *-2*. This finding is consistent with our previous observation that all components of known TGFbeta signaling pathways are present in these cell lines [28]. Another 17 Venn-restricted terms comprised other growth factors, of which 8 were expressed in at least one of the cell lines (Figure 9B). While not included in the Venn-restricted data set, we also assessed the presence of Wnt family members and BMP antagonists (Figure 9C) since these signaling pathways have been extensively implicated in the early phases of cardiac specification. We found that all of the cell lines expressed the BMP antagonists *Follistatin* and *Noggin*, but only three Wnt family members, *Wnt11*, *Wnt4* and *Wnt7b* were expressed. Finally, of the 175 remaining Venn-restricted terms, 38 were expressed in at least one of the cell lines (Figure 10). These data represent a comprehensive set of secreted factors with known receptor binding activity that are expressed in cells lines possessing heart inducing ability.

We observed marked differences between END2 cells and XEN cells in terms of their effects on cardiac development. XEN-CM was the only CM that enhanced beating when added prior to mesoderm formation and was the only CM to maintain enhanced expression of cardiac markers until day 10. By contrast, END2-CM was the only CM to block, or delay, cardiomyocyte formation when added prior to mesoderm formation. Because of this, we performed further analysis to identify the genes that were most upregulated in END2 cells as compared to PYS2 and XEN cell lines (Table S1, END2 cells and Table S2, XEN cells). Top hits were compared using the DAVID bioinformatics tool (http://david.abcc.ncifcrf.gov/) [44], [45] to determine molecular functions or pathways unique to these cells that might account for their distinct functions. Analysis of genes uniquely upregulated in XEN cells revealed a number of factors known to be involved in heart development (p-value 6.8E-4), calcium homeostasis (p-value 1.1E-2) or ion transport (1E-1) (Table S2). In addition, pathway analysis revealed that only XEN cells expressed the retinoic acid (RA)-degrading enzyme Cyp26a1 [46], [47], [48].

### VE formation on the surface of EBs may reveal a requirement for contact-dependent signals from the endoderm

Although addition of CM increases both the percentage of EBs that formed beating areas and the percentage of *MHCα* expressing cells in our culture system, it is equally clear that EBs spontaneously form beating cardiomyocytes without the addition of CM. Since mouse EBs generate a surface layer of extraembryonic endoderm, which resembles VE [49], we surmised that if EBs formed VE in our culture system, then some of the effects resulting from CM treatment could be masked. Conversely, it is possible that factors present in the CM simply supplement factors that are already present in the EBs.

**Figure 11:**
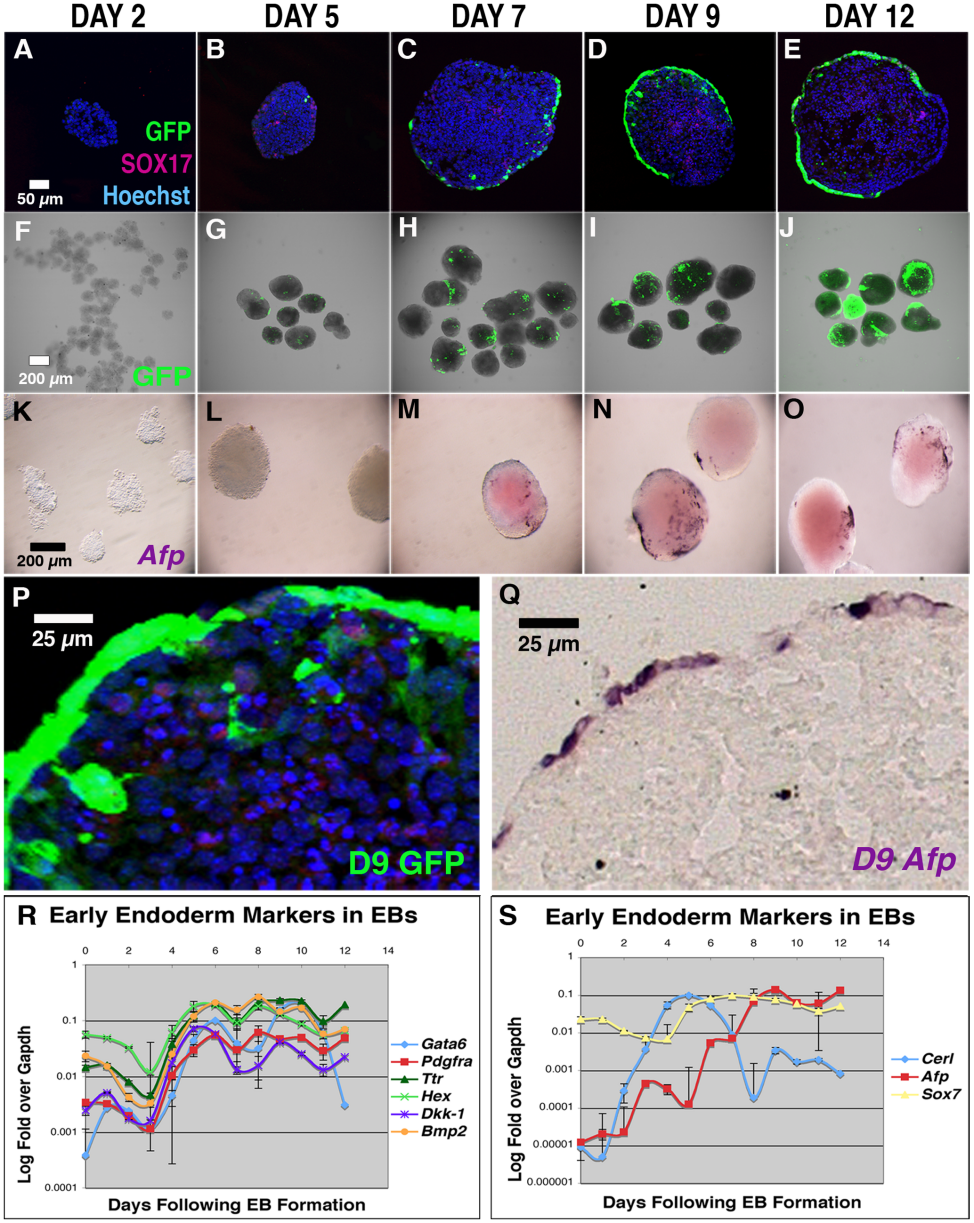
EBs form VE under conditions that promote cardiomyocyte differentiation. **A–E**. Cross-sections of EBs showing expression of the *Afp*::GFP VE reporter as well as the endodermal marker SOX17 (purple). **F–J**. Whole mount expression of *Afp*::GFP in EBs over a time course. **K–O**. Whole mount *in situs* of EBs showing expression of the endogenous *Afp*. **P**. Detail of Figure 11D. **Q**. Detail of Figure 11N. **R**. Dynamic expression of early endoderm markers (*Gata6*, *Pdgfrα*, *Ttr*, *Hex*, *Dkk-1*, *Bmp2*) over a time course. **S**. Dynamic expression of early endoderm markers (*Cerl*, *Afp*, *and Sox7*) over a time-course.

To address this question, we derived an ES cell line from a strain of mice expressing GFP under the control of the *Afp* promoter [50]. During early embryonic development, this promoter drives GFP expression in the VE. If VE forms, then we would expect to see GFP expressing cells at the surface of differentiating EBs and, starting at day 3, we could visualize GFP-positive cells on the surface of EBs. These cells eventually came to occupy the entire surface of the EB (Figure 11A–J). Although we also noted GFP-positive cells inside the EB at day 9, these internal GFP-positive cells did not express *Afp* mRNA (compare Figure 11P to Figure 11Q) and likely represented cells that had downregulated *Afp* expression but remained GFP-positive due to perdurance of the GFP protein.

We also analyzed the differentiating EBs for a panel of markers expressed in the VE (*Sox7*, *Ttr*, *Pdgfrα and Gata6*) and AVE (*Cerl*, *Hex and Dkk-1*) of mouse embryos using qRT-PCR. We found that these genes were transiently expressed, beginning at about day 3 and persisting throughout the course of the experiment (Figure 11R). Three VE markers exhibited unique patterns of expression that might indicate that they play specific roles in the formation of the VE. *Sox7* was expressed at high levels throughout, while expression of *Afp* and *Cerl* began to increase a day earlier than other markers. In addition, *Cerl* exhibited a significant downregulation by day 7, not seen with the other markers (Figure 11S). These data demonstrate that when EBs are differentiated under optimal conditions for cardiomyocyte differentiation they also make ample quantities of VE. Therefore, while there are clearly contact-independent factors secreted by the VE that enhance myocardial differentiation, our data cannot rule out the possibility that there are additional contact-dependent effects of VE as well.

### Both contact-dependent and contact-independent factors are likely to impact cardiomyocyte differentiation in EBs

**Figure 12:**
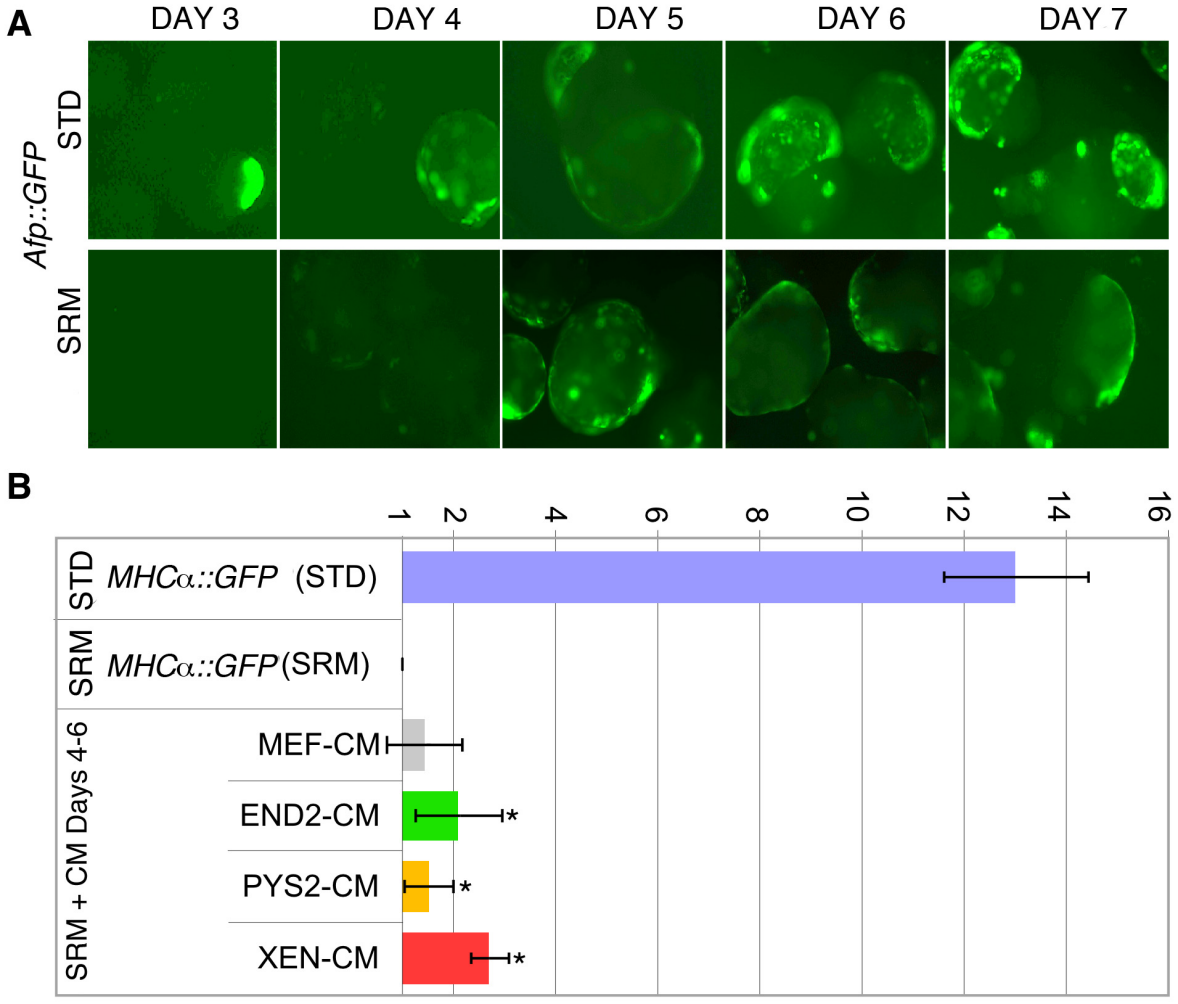
Culture conditions that delay VE formation also block cardiac differentiation, but cardiac differentiation is partially rescued by the addition of endodermally conditioned medium. **A**. Pseudo-colored fluorescence images showing *Afp*::GFP expression (Green) during EB formation under normal serum-containing conditions (STD) and during serum-free conditions (SRM). Note that VE formation is delayed by approximately 2 days. **B**. Summary of Flow Cytometry data for *MHCα*::GFP at day 10 comparing EBs grown under standard condition to those grown under serum free conditions. Addition of all three endodermally conditioned media, but not MEF conditioned medium, had a positive impact on cardiomyocyte formation.

To address the possibility that VE formation within the EB may mask the requirement for contact-dependent signals in cardiomyocyte formation, we empirically determined conditions in which VE formation within EBs was delayed (Figure 12A). Briefly, EB size was decreased and serum was replaced with a defined serum replacement. Under these conditions, we found that VE formation began approximately 2 days later than in controls. In this condition we also found that mesoderm formed robustly (as assessed by the expression of *Brachyury*), but that cardiac differentiation was severely impaired with only a small background of cells expressing the *MHCα*::*GFP* reporter and beating (data not shown). To test whether factors in the CM could rescue cardiac differentiation, we added CMs (also produced in serum-free media) at days 4–6 of EB differentiation (Figure 12B). At day 10 we analyzed the percentage of cells within the culture that underwent myocardial differentiation and noted a 2–3 fold increase in cardiomyocyte differentiation when EBs were treated with CM as compared to EBs grown in the serum-free media alone. However, the total number of myocardial cells that formed remained lower than those that formed under standard, serum containing, media conditions. This suggests that there are either contact-dependent signals produced by the VE or that there are factors in serum, but absent in serum-free media that are required for expansion of cardiac progenitors that form within EBs.

## Discussion

### Identifying the heart inducing factors produced by the AVE

Heart disease is a leading cause of death and decline among adults in much of the developed world. In particular, ventricular infarct is highly correlated with poor medical outcome. For this reason, much effort has been directed toward the identification of possible sources of donor myocardial cells that could be used to repair damaged tissue. This might be accomplished either by the *in vitro* differentiation of cardiomyocytes from stem cell sources or the *in vivo* mobilization and directed differentiation of stem cells resident in the heart or other adult tissues.

Efforts to differentiate cardiomyocytes *in vitro* have been hampered by the observation that while mouse and some human ES cells readily become cardiomyocytes when differentiated in EBs, this ability to form cardiomyocytes varies between cell lines. The reason for this is unclear, but our data suggest that this could be due to visceral endoderm (VE) formation in EBs. Here, we show that conditions promoting myocardial differentiation also favor VE formation and that conditions that delay or block VE formation also block heart formation. In support of our observations, both human ES cells and mouse epiblast stem cells (EpiSCs) display the propensity to form cardiomyocytes, which correlates with a propensity to form extraembryonic endoderm [51], [52]. Further support for this idea comes from a recently devised protocol in which the differentiation of human ES cells toward cardiomyocyte lineages is greatly enhanced as compared to untreated human ES cells. [53]. In this protocol, EBs were treated with a cocktail of BMP and Activin, the latter of which mimics the effects of Nodal. Six days after treatment, a distinct KDR^lo^/cKit^neg^ cell population, can be isolated by FACS. This KDR^lo^/cKit^neg^ population is highly enriched for cardiac cells, as compared to the unsorted ES cell pool [53]. While this study did not explicitly investigate VE formation, previous studies suggest that Nodal acts indirectly in heart formation by inducing VE and upregulating markers specific for the heart-inducing anterior visceral endoderm (AVE) [14].

If, as these data suggest, VE formation generally and AVE formation specifically are required in human and mouse EBs for myocardial differentiation, then understanding the signals that regulate AVE formation and the specific signals within the AVE that mediate heart formation will be critical to the development of refined protocols for myocardial differentiation. Furthermore, the identification of heart inducing factors secreted by VE-like cell lines will be critical for the molecular identification of endogenous cardiac inducing signals.

### Signals from primitive endoderm stem cells (XEN cells) enhance myocardial differentiation

XEN cells are a recently reported stem cell type isolated from blastocyst stage mouse embryos with the potential to differentiate into extraembryonic endodermal derivatives [27]. This, in combination with the fact that they can be derived from mutant strains, make them a potentially powerful tool both for the *in vitro* study of VE differentiation and for the identification of cardiogenic factors produced in the VE. Here we present the first functional characterization of the ability of XEN cells to support myocardial differentiation. We have also compared the gene expression profile of XEN cells to that of other VE cell lines, which have previously been reported to promote cardiac differentiation [15], [22], [23], [54]. Our data identify a discrete set of secreted factors that could account for the ability of VE cell types to support myocardial differentiation.

### The role of retinoic acid in heart formation

In these studies, only XEN-CM influenced whether or not an EB formed a beating area when added prior to mesoderm formation. The fact that XEN-CM affected the acquisition of beating, but did not affect the subsequent differentiation or proliferation of cardiomyocytes when added in this time window, suggests that signals from XEN-CM affect a very early step in the cardiomyocyte differentiation program. Indeed, when XEN-CM was added in this early time window, we noted an increase in the expression of *Fgf8*, which is required in the mouse for the establishment of cardiac progenitors [55], [56]. However, most cardiac markers remained unchanged or were downregulated as compared to controls. One factor that was differentially expressed by XEN cells, as compared to the other heart inducing extraembryonic endoderm cell lines, was the retinoic acid (RA) degrading enzyme *Cyp26a1* [48], [57], [58], thereby implicating RA signaling in this process.

A large body of data suggests that there is an antagonistic relationship between RA and FGF signaling in the neural plate [59], [60], in the heart [61] and in the primitive streak/tail bud [62], [63]. Notably, RA signaling has also been show to restrict the size of the cardiac progenitor pool in zebrafish [64], and to block myocardial differentiation in *Xenopus* [65]. The role of RA in activating heart formation from mouse EBs is unclear, since both RA [66] and RA antagonists [67] have been reported to increase myocardial differentiation of ES cells. These differences could be related to nuances in the protocols used or could reflect issues of cardiac patterning since different markers were used to assess cardiac differentiation. Nonetheless, our findings, together with these earlier studies suggest that RA signaling is likely to play complex roles in the induction and patterning of the myocardium.

### Insight into the role of Cerberus in heart formation

One unexpected observation from the current study relates to the expression and function of *Cerl*, a marker of the AVE in mouse embryos. We had previously shown that activation of *Cerberus* in the endoderm downstream of Nodal signaling is required for normal cardiac induction and is sufficient to activate ectopic heart field formation in *Xenopus* embryos [14]. Our present analysis of marker expression in differentiating EBs suggests that *Cerl* is activated earlier during EB differentiation than other VE markers (Figure 11S), suggesting a possible functional role in establishing the AVE. Despite the apparent importance of *Cerl* in heart induction, none of the heart inducing cell lines express this marker (Figure 9C). Since active *Cerl* expression is not required for the heart inducing ability of VE cell lines, *Cerl* may act transiently in the VE to activate other AVE markers.

### Contact-dependent and contact-independent roles for endoderm

Our findings suggest that extraembryonic endodermal cell lines express factors capable of initiating and/or enhancing myocardial differentiation of mouse ES cells. The fact that this effect can be mediated by conditioned media (CM), as opposed to co-culture, suggests that the heart inducing effect of the VE is independent of cell contact. On the other hand, our data demonstrate a similarly important role for contact-dependent effects of endoderm in heart formation. In culture conditions that delay VE formation we find that heart induction is greatly reduced and that this reduction is only partially rescued by the addition of extraembryonic endodermal CM. Thus, factors from CM may serve to supplement required contact-dependent signals from the VE. Future studies aimed at addressing this question will necessitate the generation of EBs lacking VE or the conditional ablation of VE from established EBs.

## Materials and methods

### Ethics Statement

Since this work does not include human participants, these studies do not require institutional review board approval. Animal studies in which the *Afp*:*GFP* ES cell line was derived, were carried out with IACUC approvals.

### Cell Culture

Mouse *Afp*::*GFP* ES cells were derived from hemizygous blastocysts possessing the *Afp*::*GFP* reporter as described in Kwon *et al.* [50] using standard procedures for ES cell derivation [68]. CGR8-*MHCα*-IRES::*GFP* [30] and *Afp*::*GFP* ES cells were maintained in ES cell growth medium comprising high glucose Dulbecco's Modified Eagles Medium (DMEM) (Mediatech) supplemented with 10% ES qualified Fetal Bovine Serum (GIBCO), non-essential amino acids (Mediatech), L-glutamine, sodium pyruvate (Mediatech), LIF (Millipore), Penicillin/Streptomycin (Mediatech) and β-mercaptoethanol (Sigma). XEN cells were derived from ICR strain blastocyst stage embryos according to standard procedures [27]. END2 cells were derived from P19 embryonal carcinoma cell lines [69] and PYS2 cells were derived from 129 strain mice tumor cells [70]. XEN and PYS2 cells were both maintained in ES medium without LIF. END2 cells were grown in DMEM/F12 1∶1 media (Mediatech) supplemented with 10% FBS, L-glutamine, non-essential amino acids and Penicillin/Streptomycin (Mediatech). Media conditioned by 24-hour incubations with each of the three cell lines (XEN, PYS2 or END2) were collected, sterile filtered and stored at −20°C. These CMs are referred to as XEN-CM, PYS2-CM and END2-CM, respectively.

### EB Differentiation

*CGR8-MHCα-IRES::GFP* and *Afp::GFP* ES cells were differentiated in standard differentiation medium, which is comprised of Iscove's Modified Dulbecco's Medium (IMDM) (Gibco), supplemented with 10% differentiation grade FBS (Gibco), 0.5 mM ascorbic acid, 4.5×10−4 M monothioglycerol (Sigma), 5% protein-free Hybridoma Media (PFHM-II) (Gibco), 200 µg/mL apo-Transferrin (Sigma) and Penicillin/Streptomycin (100 units/mL and 100 µg/mL, respectively) (Mediatech). EBs were formed in 20 µL hanging drops (each comprising 300 cells) and incubated at 37°C for 24 hours. EBs were then washed into Petri dishes (Fisher) or re-plated onto 0.1% gelatin coated tissue culture dishes.

For differentiation in the absence of FBS, serum-free differentiation medium was made with Knockout® DMEM (Gibco). This medium was supplemented with 20% Knockout Serum Replacement (Gibco), 1× non-essential amino acids (Mediatech), 1× L-glutamine (Mediatech), 0.1mM β-mercaptoethanol (Sigma), 9.6 ml 7.5% sodium bicarbonate, and 6ml nucleotide solution. Nucleotide solution was reconstituted as follows: 80mg adenosine, 73mg cytodine, 85 mg guanine, 73mg uridine, and 24mg thymidine (all from Sigma) per 100mL of cell culture grade water [71].

At the onset of serum-free differentiation, EBs were made in standard differentiation medium (with FBS) as previously described. Importantly, however, 20µl hanging drops were made with 100 cells (i.e. 100 cells/EB) instead of 300cells/EB. EBs were then washed into serum-free differentiation medium on day 2 of differentiation, and media was changed every 2 days.

### Real Time PCR

EBs were collected on specific days of differentiation, RNA was isolated using Tri Reagent (Sigma), and cDNA was transcribed using Quantitect Reverse Transcription Kit (Qiagen). qRT-PCR reactions were carried out using 50 ng template/reaction in SybrGreen Master Mix (Roche, cat #: 04707516001), on a Roche LightCycler ® 480 Real-Time PCR Instrument, and analyzed with the LightCycler 480 software package (version 1.5.0.39). Crossing point data were first adjusted to reflect the efficiency of primer pairs by comparison to standard curves, (based on dilution series over a total dynamic range of 1∶1,000 or 1∶10,000 for positive control cDNAs). These data were then normalized to the ubiquitously expressed mRNA *Gapdh*. Finally, data were re-normalized to untreated controls. Significance was determined by comparison of treated and untreated samples by *t*-test. Changes were considered significant if the *p*-value was less than 0.05. In all graphs, error bars reflect standard error calculated from 3–4 separate trials.

The primers used in this study are as follows:

*Alpha-fetoprotein* (*Afp*): forward AGCTGACAACAAGG GGAGTG, reverse TTAATAATGGTTGTTGCCTGGA; *Brachyury*: forward AGCTTCGTGACGGCTGACAA, reverse CGAGTCTGGGTGGATGTAG; *Cerberus-like (Cerl)*: forward GCAGACCTATGTGTGGA, reverse ATGAGACATGATCGCTTT; *Bmp2*: forward TGTGGGCCCTCATAAAGAAGC, reverse AGGGTGCAGGCAGGAAACATA; *Dkk-1*: forward TACAATGATGGCTCTCTGCAGCCT, reverse TGGTCAGAGGGCATGCATATTCCA; *Fgf8*: forward GCTCATTGTGGAGACCGATAC, reverse TTGCTCTTGGCAATTAGCTTC; *Foxa2*: forward CGGCCAGCGAGTTAAAGTAT, reverse TCATGTTGCTCACGGAAGAG; *Gapdh*: forward AATGGATACGGCTACAGC, reverse GTGCAGCGAACTTTATTG; *Gata4*: forward CATCAAATCGCAGCCT, reverse AAGCAAGCTAGAGTCCT; *Gata6*: forward CGGGCGCAGGCAGTGTGAGT, reverse CCAAGCCGCCGTGATGAAGG; *Hex*: forward GGAGGCTGATCTTGACT, reverse GTAGGGACTGCGTCAT; *Islet 1* (*Isl1*): forward GAGTCATCCGAGTGTGGTTTC, reverse ACCATGGGAGTTCCTGTCATC; *Mesp-1*: forward AATGCAACGGATGATTGT, reverse AGCGTGTACCCTATTGG; *Myosin Heavy Chain-alpha* (*MHCα*): forward CATGCCAATGACGACCT, reverse CCTACACTCCTGTACTGCC; *Myosin Heavy Chain-beta* (*MHCβ*): forward GGTGGCAAAGTCACTGCTGA, reverse ACAGGCAGCCACTTGTAGGG; *Nkx2.5*: forward TTACCGGGAGCCTACGGTG, reverse GCTTTCCGTCGCCGCCGTGCGCGTG; *Nppa*: forward GTGGGCAGAGACAGCAAACA, reverse TCTGTGTTGGACACCGCACT; *Shox2*: forward TCCCCTGAACTGAAGGATCG, reverse CAGTCGCTGGCTCAATTCCT *Tbx5*: forward CCAGCTCGGCGAAGGGATGTTT, reverse CCGACGCCGTGTACCGAGTGAT; *cardiac Troponin I* (*cTnI*): forward CCGCCTCCAGAAAACTTCAG, reverse CGTGAAGCTGTCGGCATAAG; *cardiac Troponin T* (*cTnT*): forward GAGGTGGTGGAGGAGTACGA, reverse GTTGGCCTCCTCTGTCTCAG.

### Flow Cytometry

EBs were collected and dissociated into single cell suspensions using 0.25% Trypsin/EDTA (Mediatech) or 2.4U/ml Dispase/2.5mg/ml Collagenase D mixture. Cells were centrifuged and re-suspended in 1% BSA-PBS then sterile filtered through an 80µm sieve. Flow cytometry was performed with a Becton-Dickinson (B–D) FACScan, and data were acquired using B–D CellQuest software.

### *In Situ* Hybridization

An *in situ* hybridization protocol for mouse embryos was modified from [72] for use on EBs. Briefly, EBs were fixed in 4% paraformaldehyde and serially dehydrated in methanol for storage. EBs were re-hydrated, washed with PBS+0.1% Tween-20 (PBT), and fixed with 4% paraformaldehyde (PFA)/0.2% Glutaraldehyde. After washing, EBs were prehybridized for 1 hour at 65°C followed by overnight hybridization with ≥1µg/mL of probe. EBs were washed with 50% Formamide/5XSSC/1% SDS at 65°C, treated with RNaseA, followed by washes with 50% Formamide/2XSSC and Tris-buffered Saline+0.1% Tween-20 (TBST). EBs were then incubated overnight at 4°C in anti-DIG-AP (1∶1000) and 1% Boehringer blocking buffer. EBs were washed extensively in TBST. Color was developed with BCIP/NBT, and once color had developed, EBs were fixed with 4% PFA. For sectioning, EBs were embedded in Tissue-Tek O.C.T. compound and 12 µm cryosections were cut on a Leica (CM3050) cryostat.

### Immunohistochemistry

EB dissociation was performed as described above for flow cytometery studies, cells were plated onto chamber slides and incubated overnight at 37°C in differentiation medium to allow cells to attach. Cells were washed briefly with PBS and fixed in 4% PFA for 15 minutes. Blocking was carried out with 3% FBS-2% BSA PBS for 1hr. Primary antibodies, anti-GFP, anti-Troponin T-C (C-19), anti-Actin (C-11) (all from Santa Cruz Biotechnology, Inc.), or anti-Sarcomeric Myosin, MF20 (Developmental Studies Hybridoma Bank), were diluted in blocking solution to concentrations appropriate to the specific antibody and added to cells. Cells were incubated overnight at 4°C, then washed with PBS and blocked for 30 minutes at room temperature. Isotype appropriate secondary antibodies, conjugated to Alexa Fluor® 488 or Alexa Fluor® 555, (Invitrogen) were diluted and added to cells, followed by an overnight incubation at 4°C. Finally, cells were washed with PBS and cover slipped with Vectashield mounting medium containing DAPI.

### Microarray Analysis

Total RNA was isolated with Qiagen RNeasy Mini Kit and used to probe Illumina expression array (MouseWG-6_V2_0_R0_11278593) in triplicate for each of three heart-inducing cell lines using Illumina BeadStudio version 3.4.0. The raw Illumina data (9 arrays) was analyzed using Bioconductor packages. The data was first normalized using LumiExpresso ( ) function. The differentially expressed genes in each pair-wise comparison were obtained using Limma ( ) R-package. For gene ontology studies, Illumina probes were mapped to gene symbol names using getAnnote.Illumina (“MouseWG-6_V2_0_R0_11278593_A.bz2”) downloaded from Bioconductor website: http://www.bioconductor.org/download.

Pathway and expression analysis was carried out using DAVID Bioinformatics Resources 2008 sponsored by the National Institute of Allergy and Infectious Diseases (NIAID), NIH, at http://david.abcc.ncifcrf.gov/ [44], [45]. This data is MIAME compliant and has been deposited in NCBI's Gene Expression Omnibus [73]. All data is accessible through GEO Series accession number GSE19564 (http://www.ncbi.nlm.nih.gov/geo/query/acc.cgi?acc=GSE1956.

## Supporting Information

Table S1 Factors specifically up-regulated in END2 cells. List of factors that are more highly expressed in END2 as compared to both PYS2 and XEN cells. Genes are highlighted based on Gene Ontology (GO) Consortium classifications. Yellow indicates: BP = Developmental Process GO: 0032502; Blue: BP = Cell Adhesion GO: 0007155; Green: both Developmental Process and Cell Adhesion.
(0.15 MB DOC)

Table S2 Factors specifically upregulated in XEN cells. List of factors that are more highly expressed in XEN cells as compared to both PYS2 and END2 cells. Genes are highlighted based on Gene Ontology (GO) Consortium classifications. Yellow: BP = Developmental Process GO: 0032502; Red: BP = Development and Heart Development GO: 0007507; Silver: BP = Calcium Homeostatsis GO: 0055074 or ion transport GO: 0006811.
(0.11 MB DOC)

Movie S1 Movie of untreated MHCα::GFP-expressing EBs at day 10 of differentiation.
(0.51 MB MOV)

Movie S2 Movie of MHCα::GFP-expressing EBs on day 10 following treatment with XEN-CM on days 4-6.
(0.46 MB MOV)

## References

[1] Nascone N, Mercola M (1995). An inductive role for the endoderm in *Xenopus* cardiogenesis.. Development.

[2] Sater AK, Jacobson AG (1990). The role of the dorsal lip in the induction of heart mesoderm in *Xenopus laevis*.. Development.

[3] Schneider VA, Mercola M (1999). Spatially distinct head and heart inducers within the Xenopus organizer region.. Curr Biol.

[4] Jacobson AG (1960). Influences of ectoderm and endoderm on heart differentiation in the newt.. Developmental Biology.

[5] Jacobson AG, Duncan JT (1968). Heart induction in salamanders.. Journal of Experimental Zoology.

[6] Fullilove SL (1970). Heart induction: distribution of active factors in newt endoderm.. J Exp Zool.

[7] Sater AK, Jacobson AG (1989). The specification of heart mesoderm occurs during gastrulation in Xenopus laevis.. Development.

[8] Arai A, Yamamoto K, Toyama J (1997). Murine cardiac progenitor cells require visceral embryonic endoderm and primitive streak for terminal differentiation.. Developmental Dynamics.

[9] Yatskievych T, Ladd A, Antin P (1997). Induction of cardiac myogenesis in avian pregastrula epiblast: the role of the hypoblast and activin.. Development.

[10] Matsui H, Ikeda K, Nakatani K, Sakabe M, Yamagishi T (2005). Induction of initial cardiomyocyte alpha-actin–smooth muscle alpha-actin–in cultured avian pregastrula epiblast: a role for nodal and BMP antagonist.. Dev Dyn.

[11] Tam PP, Parameswaran M, Kinder SJ, Weinberger RP (1997). The allocation of epiblast cells to the embryonic heart and other mesodermal lineages: the role of ingression and tissue movement during gastrulation.. Development.

[12] Foley A, Mercola M (2005). Heart induction by Wnt antagonists depends on the homeodomain transcription factor Hex.. Genes and Development.

[13] Foley AC, Gupta RW, Guzzo RM, Korol O, Mercola M (2006). Embryonic heart induction.. Ann N Y Acad Sci.

[14] Foley AC, Korol O, Timmer AM, Mercola M (2007). Multiple functions of Cerberus cooperate to induce heart downstream of Nodal.. Dev Biol.

[15] Nijmeijer RM, Leeuwis JW, DeLisio A, Mummery CL, Chuva de Sousa Lopes SM (2009). Visceral endoderm induces specification of cardiomyocytes in mice.. Stem Cell Res.

[16] Foley AC, Skromne I, Stern CD (2000). Reconciling different models of forebrain induction and patterning: a dual role for the hypoblast.. Development.

[17] Foley AC, Stern CD (2001). Evolution of vertebrate forebrain development: how many different mechanisms?. J Anat.

[18] Gadue P, Huber TL, Nostro MC, Kattman S, Keller GM (2005). Germ layer induction from embryonic stem cells.. Exp Hematol.

[19] Boheler KR, Czyz J, Tweedie D, Yang HT, Anisimov SV (2002). Differentiation of pluripotent embryonic stem cells into cardiomyocytes.. Circ Res.

[20] Wei H, Juhasz O, Li J, Tarasova YS, Boheler KR (2005). Embryonic stem cells and cardiomyocyte differentiation: phenotypic and molecular analyses.. J Cell Mol Med.

[21] Wobus AM, Boheler KR (2005). Embryonic stem cells: prospects for developmental biology and cell therapy.. Physiol Rev.

[22] Mummery C, Ward-van Oostwaard D, Doevendans P, Spijker R, van den Brink S (2003). Differentiation of human embryonic stem cells to cardiomyocytes: role of coculture with visceral endoderm-like cells.. Circulation.

[23] Passier R, Oostwaard DW, Snapper J, Kloots J, Hassink RJ (2005). Increased cardiomyocyte differentiation from human embryonic stem cells in serum-free cultures.. Stem Cells.

[24] Mummery C, Ward D, van den Brink CE, Bird SD, Doevendans PA (2002). Cardiomyocyte differentiation of mouse and human embryonic stem cells.. J Anat.

[25] Stary M, Pasteiner W, Summer A, Hrdina A, Eger A (2005). Parietal endoderm secreted SPARC promotes early cardiomyogenesis in vitro.. Exp Cell Res.

[26] Holtzinger A, Rosenfeld GE, Evans T (2010). Gata4 directs development of cardiac-inducing endoderm from ES cells.. Dev Biol.

[27] Kunath T, Arnaud D, Uy GD, Okamoto I, Chureau C (2005). Imprinted X-inactivation in extra-embryonic endoderm cell lines from mouse blastocysts.. Development.

[28] Brown K, LeGros S, Artus J, Doss MX, Khanin R (2010). A comparative analysis of Extra-embryonic Endoderm Cell lines.. PLoS One.

[29] Lim CY, Tam WL, Zhang J, Ang HS, Jia H (2008). Sall4 regulates distinct transcription circuitries in different blastocyst-derived stem cell lineages.. Cell Stem Cell.

[30] Takahashi T, Lord B, Schulze PC, Fryer RM, Sarang SS (2003). Ascorbic acid enhances differentiation of embryonic stem cells into cardiac myocytes.. Circulation.

[31] Yasunaga M, Tada S, Torikai-Nishikawa S, Nakano Y, Okada M (2005). Induction and monitoring of definitive and visceral endoderm differentiation of mouse ES cells.. Nat Biotechnol.

[32] Saga Y, Miyagawa-Tomita S, Takagi A, Kitajima S, Miyazaki J (1999). MesP1 is expressed in the heart precursor cells and required for the formation of a single heart tube.. Development.

[33] Franco D, Lamers WH, Moorman AF (1998). Patterns of expression in the developing myocardium: towards a morphologically integrated transcriptional model.. Cardiovasc Res.

[34] Bruneau BG, Logan M, Davis N, Levi T, Tabin CJ (1999). Chamber-specific cardiac expression of Tbx5 and heart defects in Holt-Oram syndrome.. Dev Biol.

[35] Liberatore CM, Searcy-Schrick RD, Yutzey KE (2000). Ventricular expression of tbx5 inhibits normal heart chamber development.. Dev Biol.

[36] Biben C, Harvey RP (1997). Homeodomain factor Nkx2-5 controls left/right asymmetric expression of bHLH gene eHand during murine heart development.. Genes Dev.

[37] Thomas T, Yamagishi H, Overbeek PA, Olson EN, Srivastava D (1998). The bHLH factors, dHAND and eHAND, specify pulmonary and systemic cardiac ventricles independent of left-right sidedness.. Dev Biol.

[38] Christoffels VM, Habets PE, Franco D, Campione M, de Jong F (2000). Chamber formation and morphogenesis in the developing mammalian heart.. Developmental Biology.

[39] Gourdie RG, Severs NJ, Green CR, Rothery S, Germroth P (1993). The spatial distribution and relative abundance of gap-junctional connexin40 and connexin43 correlate to functional properties of components of the cardiac atrioventricular conduction system.. J Cell Sci.

[40] Cai CL, Liang X, Shi Y, Chu PH, Pfaff SL (2003). Isl1 identifies a cardiac progenitor population that proliferates prior to differentiation and contributes a majority of cells to the heart.. Dev Cell.

[41] Espinoza-Lewis RA, Yu L, He F, Liu H, Tang R (2009). Shox2 is essential for the differentiation of cardiac pacemaker cells by repressing Nkx2-5.. Dev Biol.

[42] Gannon M, Bader D (1995). Initiation of cardiac differentiation occurs in the absence of anterior endoderm.. Development.

[43] Ashburner M, Ball CA, Blake JA, Botstein D, Butler H (2000). Gene ontology: tool for the unification of biology. The Gene Ontology Consortium.. Nat Genet.

[44] Dennis G, Sherman BT, Hosack DA, Yang J, Gao W (2003). DAVID: Database for Annotation, Visualization, and Integrated Discovery.. Genome Biol.

[45] Huang da W, Sherman BT, Lempicki RA (2009). Systematic and integrative analysis of large gene lists using DAVID bioinformatics resources.. Nat Protoc.

[46] Abu-Abed S, Dolle P, Metzger D, Beckett B, Chambon P (2001). The retinoic acid-metabolizing enzyme, CYP26A1, is essential for normal hindbrain patterning, vertebral identity, and development of posterior structures.. Genes Dev.

[47] Abu-Abed SS, Beckett BR, Chiba H, Chithalen JV, Jones G (1998). Mouse P450RAI (CYP26) expression and retinoic acid-inducible retinoic acid metabolism in F9 cells are regulated by retinoic acid receptor gamma and retinoid X receptor alpha.. J Biol Chem.

[48] White JA, Beckett-Jones B, Guo YD, Dilworth FJ, Bonasoro J (1997). cDNA cloning of human retinoic acid-metabolizing enzyme (hP450RAI) identifies a novel family of cytochromes P450.. J Biol Chem.

[49] Artus J, Panthier JJ, Hadjantonakis AK (2010). A role for PDGF signaling in expansion of the extra-embryonic endoderm lineage of the mouse blastocyst.. Development.

[50] Kwon GS, Fraser ST, Eakin GS, Mangano M, Isern J (2006). Tg(Afp-GFP) expression marks primitive and definitive endoderm lineages during mouse development.. Dev Dyn.

[51] Pal R, Totey S, Mamidi MK, Bhat VS, Totey S (2009). Propensity of human embryonic stem cell lines during early stage of lineage specification controls their terminal differentiation into mature cell types.. Exp Biol Med (Maywood).

[52] Tesar PJ, Chenoweth JG, Brook FA, Davies TJ, Evans EP (2007). New cell lines from mouse epiblast share defining features with human embryonic stem cells.. Nature.

[53] Yang L, Soonpaa MH, Adler ED, Roepke TK, Kattman SJ (2008). Human cardiovascular progenitor cells develop from a KDR(+) embryonic-stem-cell-derived population.. Nature.

[54] Mummery CL, van Achterberg TA, van den Eijnden-van Raaij AJ, van Haaster L, Willemse A (1991). Visceral-endoderm-like cell lines induce differentiation of murine P19 embryonal carcinoma cells.. Differentiation.

[55] Ilagan R, Abu-Issa R, Brown D, Yang YP, Jiao K (2006). Fgf8 is required for anterior heart field development.. Development.

[56] Park EJ, Ogden LA, Talbot A, Evans S, Cai CL (2006). Required, tissue-specific roles for Fgf8 in outflow tract formation and remodeling.. Development.

[57] Abu-Abed M, Turner MA, Vallee F, Simpson A, Slingsby C (1997). Structural comparison of the enzymatically active and inactive forms of delta crystallin and the role of histidine 91.. Biochemistry.

[58] Liu L, Gudas LJ (2005). Disruption of the lecithin:retinol acyltransferase gene makes mice more susceptible to vitamin A deficiency.. J Biol Chem.

[59] Diez del Corral R, Olivera-Martinez I, Goriely A, Gale E, Maden M (2003). Opposing FGF and retinoid pathways control ventral neural pattern, neuronal differentiation, and segmentation during body axis extension.. Neuron.

[60] Sirbu IO, Duester G (2006). Retinoic-acid signalling in node ectoderm and posterior neural plate directs left-right patterning of somitic mesoderm.. Nat Cell Biol.

[61] Sirbu IO, Zhao X, Duester G (2008). Retinoic acid controls heart anteroposterior patterning by down-regulating Isl1 through the Fgf8 pathway.. Dev Dyn.

[62] Goldbeter A, Gonze D, Pourquie O (2007). Sharp developmental thresholds defined through bistability by antagonistic gradients of retinoic acid and FGF signaling.. Dev Dyn.

[63] Diez del Corral R, Storey KG (2004). Opposing FGF and retinoid pathways: a signalling switch that controls differentiation and patterning onset in the extending vertebrate body axis.. Bioessays.

[64] Keegan BR, Feldman JL, Begemann G, Ingham PW, Yelon D (2005). Retinoic acid signaling restricts the cardiac progenitor pool.. Science.

[65] Drysdale TA, Patterson KD, Saha M, Krieg PA (1997). Retinoic acid can block differentiation of the myocardium after heart specification.. Developmental Biology.

[66] Wobus AM, Kaomei G, Shan J, Wellner MC, Rohwedel J (1997). Retinoic acid accelerates embryonic stem cell-derived cardiac differentiation and enhances development of ventricular cardiomyocytes.. J Mol Cell Cardiol.

[67] Honda M, Hamazaki TS, Komazaki S, Kagechika H, Shudo K (2005). RXR agonist enhances the differentiation of cardiomyocytes derived from embryonic stem cells in serum-free conditions.. Biochem Biophys Res Commun.

[68] Nagy A, Gertsenstein M, Vintersten K, Behringer RR (2003). Manipulating the Mouse Embryo: A Laboratory Manual.

[69] Mummery CL, Feijen A, van der Saag PT, van den Brink CE, de Laat SW (1985). Clonal variants of differentiated P19 embryonal carcinoma cells exhibit epidermal growth factor receptor kinase activity.. Dev Biol.

[70] Lehman JM, Speers WC, Swartzendruber DE, Pierce GB (1974). Neoplastic differentiation: characteristics of cell lines derived from a murine teratocarcinoma.. J Cell Physiol.

[71] Gissel C, Doss M, Hippler-Altenburg R, Heschler J, Sachinidis A, Turksen K Methods in Molecular Biology.

[72] Wilkinson DG, Bhatt S, Herrmann BG (1990). Expression pattern of the mouse T gene and its role in mesoderm formation.. Nature.

[73] Edgar R, Domrachev M, Lash AE (2002). Gene Expression Omnibus: NCBI gene expression and hybridization array data repository.. Nucleic Acids Res.

