# Supplementary material for: eXtraembryonic ENdoderm (XEN) Stem Cells Produce Factors that Activate Heart Formation
Source: PLoS One. 2010 Oct 20;5(10):e13446. doi: 10.1371/journal.pone.0013446 (PMC2958120; doi:10.1371/journal.pone.0013446)
Supplement: Table S1 — Factors specifically up-regulated in END2 cells. List of factors that are more highly expressed in END2 as compared to both PYS2 and XEN cells. Genes are highlighted based on Gene Ontology (GO) Consortium classifications. Yellow indicates: BP = Developmental Process GO: 0032502; Blue: BP = Cell Adhesion GO: 0007155; Green: both Developmental Process and Cell Adhesion. (0.15 MB DOC) [file pone.0013446.s001.doc]

Table 1: Genes up in END2 vs. XEN and END2 vs. PYS2

| Gene_ID | Accession | Symbol | logFC1  END2 vs XEN | logFC2  END2 vs PYS2 | P.value1 | P.value2 |
| --- | --- | --- | --- | --- | --- | --- |
| 6520315 | NM_008581 | Mela | 7.2632 | 7.2509 | 3.01E-15 | 3.05E-15 |
| 4250121 | NM_175074.1 | Hmgn3 | 4.2088 | 4.2437 | 2.19E-13 | 2.04E-13 |
| 6560487 | NM_011349.2 | Sema3f | 4.4123 | 4.3921 | 7.55E-13 | 7.84E-13 |
| 4150739 | NM_026514 | Cdc42ep3 | 4.0180 | 4.5986 | 8.84E-13 | 2.82E-13 |
| 2140450 | NM_024170 | 1110012O05Rik | 3.8679 | 4.1784 | 1.03E-12 | 5.36E-13 |
| 4760424 | NM_198612.1 | BC049816 | 3.8805 | 3.9153 | 1.73E-12 | 1.60E-12 |
| 6940142 | NM_013512 | Epb4.1l4a | 3.9310 | 2.6122 | 2.25E-12 | 7.09E-11 |
| 160066 | NM_021491.2 | Smpd3 | 3.4522 | 3.4237 | 2.81E-12 | 3.01E-12 |
| 7570609 | NM_010923 | Nnat | 3.3658 | 3.3664 | 3.46E-12 | 3.46E-12 |
| 4150386 | NM_015734.1 | Col5a1 | 3.7886 | 3.4381 | 3.96E-12 | 8.99E-12 |
| 870670 | NM_008788.1 | Pcolce | 3.2131 | 2.0661 | 4.44E-12 | 1.84E-10 |
| 3290554 | XM_140952.5 | 5930402A21 | 3.4226 | 3.1332 | 4.82E-12 | 1.02E-11 |
| 1410543 | NM_010728.1 | Lox | 3.3264 | 3.3227 | 5.66E-12 | 5.71E-12 |
| 3520546 | NM_010129.1 | Emp3 | 3.5188 | 3.7678 | 1.11E-11 | 6.25E-12 |
| 6280189 | NM_028734.2 | Steap2 | 2.4607 | 2.6162 | 1.28E-11 | 7.63E-12 |
| 7550112 | NM_013655.2 | Cxcl12 | 6.3436 | 6.3012 | 1.60E-11 | 1.69E-11 |
| 670133 | NM_175074.1 | Hmgn3 | 4.4459 | 4.4764 | 2.10E-11 | 1.98E-11 |
| 4610072 | NM_007616.2 | Cav1 | 5.3127 | 5.2995 | 3.28E-11 | 3.35E-11 |
| 4220673 | XM_147847.4 | Spata13 | 3.7493 | 3.6470 | 3.65E-11 | 4.61E-11 |
| 7160167 | NM_010128.3 | Emp1 | 4.9227 | 4.5048 | 4.47E-11 | 9.45E-11 |
| 3800634 | NM_008909.2 | Ppl | 2.6296 | 2.5695 | 4.91E-11 | 5.96E-11 |
| 6380082 | NM_175126.3 | Zcchc3 | 2.8117 | 2.8132 | 8.58E-11 | 8.54E-11 |
| 1660703 | NM_144783.1 | Wt1 | 5.3245 | 5.6464 | 8.95E-11 | 5.46E-11 |
| 4150403 |  | 5830411I20 | 2.6465 | 2.6940 | 9.37E-11 | 8.07E-11 |
| 6250463 | NM_007736 | Col4a5 | 3.7203 | 3.7212 | 1.05E-10 | 1.05E-10 |
| 5670634 |  | Synpo | 4.6317 | 4.2683 | 1.11E-10 | 2.21E-10 |
| 10279 | NM_007742.2 | Col1a1 | 3.6447 | 3.6218 | 1.15E-10 | 1.21E-10 |
| 3460307 | NM_173779.2 | A630014H24Rik | 1.9750 | 1.9833 | 1.36E-10 | 1.31E-10 |
| 1170278 | NM_018764.1 | Pcdh7 | 2.6012 | 2.5997 | 1.41E-10 | 1.42E-10 |
| 5810470 | NM_013467 | Aldh1a1 | 3.8029 | 3.8712 | 1.63E-10 | 1.40E-10 |
| 1570291 | NM_198861.1 | BC046404 | 4.2605 | 3.1792 | 1.64E-10 | 1.92E-09 |
| 6330053 | NM_028238.5 | Rab38 | 2.7155 | 2.7114 | 1.74E-10 | 1.76E-10 |
| 4010224 | NM_207655.1 | Egfr | 3.9472 | 3.9825 | 1.80E-10 | 1.67E-10 |
| 580433 | XM_355338.1 | Hoxd8 | 1.8698 | 1.8698 | 1.99E-10 | 1.99E-10 |
| 7320739 | NM_175271 | Gpr23 | 2.4851 | 2.4630 | 2.05E-10 | 2.21E-10 |
| 4920300 | NM_018857.1 | Msln | 1.8007 | 1.8832 | 2.19E-10 | 1.50E-10 |
| 1030435 | NM_009955.2 | Dpysl2 | 4.0029 | 4.2732 | 2.63E-10 | 1.52E-10 |
| 5420047 | NM_009988 | Cxadr | 4.2498 | 4.4677 | 3.26E-10 | 2.14E-10 |
| 1820224 | NM_008538 | Marcks | 4.5028 | 4.6607 | 3.39E-10 | 2.54E-10 |
| 7550431 | NM_134065.2 | AU040950 | 2.5906 | 2.1892 | 3.41E-10 | 1.40E-09 |
| 3390326 | NM_198861.1 | BC046404 | 3.8600 | 3.0459 | 3.46E-10 | 2.53E-09 |
| 6960730 | NM_012011.1 | Eif2s3y | 2.1098 | 2.1469 | 3.81E-10 | 3.29E-10 |
| 5550240 | NM_007670.2 | Cdkn2b | 3.1626 | 3.3465 | 3.99E-10 | 2.48E-10 |
| 3370025 | NM_008862.2 | Pkia | 2.9659 | 3.1341 | 5.08E-10 | 3.19E-10 |
| 1170706 | NM_008471.1 | Krt1-19 | 2.7733 | 2.0125 | 5.19E-10 | 7.61E-09 |
| 6660474 | NM_010460.2 | Hoxb7 | 2.7146 | 2.5408 | 5.32E-10 | 9.27E-10 |
| 3990131 | NM_033475.2 | Rab34 | 2.5970 | 3.4617 | 5.78E-10 | 5.14E-11 |
| 160451 |  | 2900078C09Rik | 2.9024 | 3.5074 | 6.47E-10 | 1.32E-10 |
| 6660201 | NM_018884.1 | Pdzrn3 | 3.6911 | 4.5541 | 6.94E-10 | 1.18E-10 |
| 520333 | NM_175309.3 | Upk3b | 6.4345 | 5.9828 | 7.15E-10 | 1.32E-09 |
| 2450523 |  | 9030024J15Rik | 2.7862 | 2.7870 | 7.61E-10 | 7.59E-10 |
| 1510270 | NM_031998.2 | Tsga14 | 2.4542 | 1.9841 | 7.71E-10 | 4.58E-09 |
| 610360 | NM_011766.2 | Zfpm2 | 2.9052 | 2.9240 | 7.91E-10 | 7.50E-10 |
| 830369 | NM_007806.1 | Cyba | 2.1220 | 2.1282 | 8.08E-10 | 7.88E-10 |
| 6650504 | NM_010221.1 | Fkbp10 | 2.7911 | 2.7358 | 8.61E-10 | 1.02E-09 |
| 4890246 | NM_009806.1 | Cask | 1.7995 | 2.7203 | 8.84E-10 | 2.73E-11 |
| 6350451 | NM_026221 | Ppfibp1 | 2.3561 | 1.8236 | 1.10E-09 | 9.38E-09 |
| 5090184 | NM_013470.1 | Anxa3 | 3.4897 | 6.7470 | 1.14E-09 | 4.42E-12 |
| 6860309 | XM_196007.3 | B230104P22Rik | 3.4605 | 3.8536 | 1.20E-09 | 4.85E-10 |
| 3870072 | NM_008610.1 | Mmp2 | 5.7380 | 5.7643 | 1.21E-09 | 1.17E-09 |
| 1850661 | NM_175126.3 | Zcchc3 | 2.3957 | 2.3634 | 1.36E-09 | 1.53E-09 |
| 7650360 | NM_029999.3 | Lbh | 2.1239 | 1.9332 | 1.39E-09 | 3.05E-09 |
| 1780025 | NM_033475.2 | Rab34 | 2.5853 | 3.3197 | 1.40E-09 | 1.71E-10 |
| 4250692 | NM_008520 | Ltbp3 | 3.3290 | 3.1205 | 1.55E-09 | 2.66E-09 |
| 2030497 | NM_029770 | Unc5b | 2.3183 | 1.8551 | 1.60E-09 | 1.03E-08 |
| 4200093 | NM_001037809.1 | Cdh3 | 2.4881 | 2.4762 | 1.62E-09 | 1.69E-09 |
| 70386 | NM_009122 | Satb1 | 3.1347 | 2.8638 | 1.69E-09 | 3.60E-09 |
| 5360300 | NM_008509.1 | Lpl | 4.4398 | 6.1695 | 1.78E-09 | 1.12E-10 |
| 6510463 | NM_133738 | Antxr2 | 2.0132 | 2.0673 | 1.89E-09 | 1.52E-09 |
| 6550044 | NM_181404.3 | D330024H06Rik | 2.4761 | 2.9679 | 1.94E-09 | 4.24E-10 |
| 7400619 | NM_024285 | Bves | 2.0797 | 2.0897 | 2.17E-09 | 2.08E-09 |
| 2470441 | NM_010460.2 | Hoxb7 | 2.0537 | 2.0332 | 2.27E-09 | 2.47E-09 |
| 4760066 | NM_025290.2 | Tsga2 | 1.7650 | 2.1690 | 2.29E-09 | 4.06E-10 |
| 6270670 | NM_175309.3 | Upk3b | 5.8862 | 5.5393 | 2.44E-09 | 4.05E-09 |
| 2230082 | XM_359211.1 | LOC386405 | 4.4791 | 4.6598 | 2.58E-09 | 1.85E-09 |
| 460594 | NM_030704.1 | Hspb8 | 2.4015 | 3.4580 | 2.62E-09 | 1.23E-10 |
| 3450180 | NM_023056.2 | 1810009M01Rik | 4.1825 | 4.1203 | 2.81E-09 | 3.19E-09 |
| 3870079 | NM_013871.2 | Mapk12 | 2.0682 | 2.0683 | 3.41E-09 | 3.41E-09 |
| 4210376 | NM_007739 | Col8a1 | 2.0591 | 2.0796 | 3.44E-09 | 3.16E-09 |
| 1110414 | NM_199200.1 | BC025575 | 2.4912 | 1.8873 | 3.69E-09 | 3.73E-08 |

Yellow: BP=Developmental Process GO: 0032502

Blue: BP= Cell Adhesion GO: 0007155

Green: both
