# Supplementary material for: eXtraembryonic ENdoderm (XEN) Stem Cells Produce Factors that Activate Heart Formation
Source: PLoS One. 2010 Oct 20;5(10):e13446. doi: 10.1371/journal.pone.0013446 (PMC2958120; doi:10.1371/journal.pone.0013446)
Supplement: Table S2 — Factors specifically upregulated in XEN cells. List of factors that are more highly expressed in XEN cells as compared to both PYS2 and END2 cells. Genes are highlighted based on Gene Ontology (GO) Consortium classifications. Yellow: BP = Developmental Process GO: 0032502; Red: BP = Development and Heart Development GO: 0007507; Silver: BP = Calcium Homeostatsis GO: 0055074 or ion transport GO: 0006811. (0.11 MB DOC) [file pone.0013446.s002.doc]

Table 2: Genes up in XEN cells vs. PYS2 and XEN vs. END2

| Gene_ID | Accession | Symbol | logFC1  XEN vs PYS2 | logFC2_  XEN vs END2 | P.value1 | P.value2 |
| --- | --- | --- | --- | --- | --- | --- |
| 2760053 | XM_486328 | 6430704N06 | 4.028 | 3.9267 | 2.45E-13 | 3.05E-13 |
| 4780286 | XM_356977.1 | LOC383308 | 4.091 | 4.3992 | 1.42E-11 | 7.67E-12 |
| 6620521 | XM_355877.1 | Kcnk6 | 3.072 | 2.7352 | 1.60E-11 | 4.28E-11 |
| 7610017 | NM_133362.1 | Erdr1 | 2.739 | 3.1258 | 1.07E-10 | 3.51E-11 |
| 6900167 | XM_144076.3 | A230053A07Rik | 2.2571 | 2.3716 | 1.87E-10 | 1.23E-10 |
| 240731 | NM_011576.1 | Tfpi | 1.5916 | 2.3322 | 2.11E-10 | 8.41E-12 |
| 4830040 | NM_176930.2 | C130076O07Rik | 2.350 | 2.4242 | 2.42E-10 | 1.87E-10 |
| 4490367 | NM_010882.2 | Ndn | 2.6426 | 2.5845 | 4.30E-10 | 5.18E-10 |
| 7380528 | NM_011196.1 | Ptger3 | 1.6985 | 1.6789 | 4.43E-10 | 4.88E-10 |
| 270736 | NM_177753.2 | Sox21 | 3.1118 | 3.1016 | 5.89E-10 | 6.06E-10 |
| 3120546 | NM_175316.2 | Slco2b1 | 3.0750 | 3.1404 | 6.50E-10 | 5.45E-10 |
| 5560112 | NM_029606.3 | Ccdc46 | 2.1556 | 1.6612 | 8.58E-10 | 7.60E-09 |
| 1580021 | NM_009082.2 | Rpl29 | 2.9982 | 3.2000 | 9.13E-10 | 5.29E-10 |
| 5890059 | NM_013514 | Epb4.9 | 1.6887 | 1.8137 | 1.86E-09 | 1.02E-09 |
| 4860392 | NM_133226.1 | Pdzk2 | 2.0242 | 2.3419 | 2.32E-09 | 6.84E-10 |
| 3390639 |  | Igsf10 | 1.3244 | 1.2940 | 3.58E-09 | 4.34E-09 |
| 5890195 | NM_022316.1 | Smoc1 | 2.4576 | 3.0679 | 4.03E-09 | 6.28E-10 |
| 5490121 | NM_007603.2 | Capn6 | 3.2633 | 3.9908 | 5.94E-09 | 1.10E-09 |
| 1230767 | NM_009378.1 | Thbd | 3.4635 | 3.8137 | 6.11E-09 | 2.73E-09 |
| 840446 | NM_178255.3 | Hapln3 | 1.6657 | 1.6458 | 6.65E-09 | 7.35E-09 |
| 2510382 | XM_194207.3 | 3732412D22Rik | 2.3653 | 2.1557 | 6.90E-09 | 1.50E-08 |
| 3400577 | NM_008653.1 | Mybpc3 | 1.6404 | 1.7346 | 7.09E-09 | 4.45E-09 |
| 2190168 | NM_023868 | Ryr2 | 1.1868 | 1.1938 | 9.13E-09 | 8.69E-09 |
| 1470044 | NM_010068.1 | Dnmt3b | 1.444 | 4.4435 | 1.00E-08 | 7.84E-13 |
| 1990551 | NM_007811.1 | Cyp26a1 | 2.3185 | 2.2860 | 1.11E-08 | 1.25E-08 |
| 50168 | NM_028455 | 3110043J09Rik | 1.5387 | 1.2280 | 1.12E-08 | 7.25E-08 |
| 3870097 | XM_484351 | 1810063B07Rik | 1.9655 | 3.0210 | 1.41E-08 | 3.87E-10 |
| 5220097 | NM_011243.1 | Rarb | 1.1491 | 1.2268 | 1.67E-08 | 9.68E-09 |
| 1500341 | NM_025920 | Thap4 | 1.1953 | 1.4089 | 2.61E-08 | 6.65E-09 |
| 3520148 | NM_199195.1 | Bckdhb | 1.4041 | 1.3730 | 2.63E-08 | 3.16E-08 |
| 1770152 | NM_010580 | Itgb5 | 1.6695 | 1.9017 | 3.29E-08 | 1.11E-08 |
| 4670246 | NM_009616 | Adam19 | 1.9010 | 3.9258 | 3.62E-08 | 8.35E-11 |
| 730138 | NM_178045.3 | Rassf4 | 1.2992 | 1.4062 | 4.27E-08 | 2.21E-08 |
| 2630446 | NM_015798.1 | Fbxo15 | 1.6942 | 3.7773 | 4.41E-08 | 5.35E-11 |
| 4200202 | XM_484234 | 6330500D04Rik | 1.5534 | 1.7393 | 4.83E-08 | 1.89E-08 |
| 3940598 | XM_127662.2 | 4933428D01Rik | 1.5750 | 1.8337 | 4.98E-08 | 1.41E-08 |
| 7320482 | XM_135109.4 | 9430077D24Rik | 1.1304 | 1.1440 | 5.14E-08 | 4.65E-08 |
| 4040095 | NM_207648 | H2-Q6 | 1.1337 | 1.2130 | 6.30E-08 | 3.60E-08 |
| 6940324 | NM_178045.3 | Rassf4 | 1.2172 | 1.2875 | 8.56E-08 | 5.38E-08 |
| 3710187 | NM_026119.2 | Vdrip | 1.4762 | 2.5035 | 8.74E-08 | 1.07E-09 |
| 3060019 |  | Fbxo15 | 1.6923 | 3.9478 | 8.76E-08 | 7.36E-11 |
| 7510722 | XM_358096.1 | LOC385163 | 1.2328 | 1.4949 | 1.10E-07 | 2.23E-08 |
| 4730445 | XM_193940.2 | Bloc1s2 | 1.0521 | 1.1853 | 1.10E-07 | 4.13E-08 |
| 2970500 | NM_148942.1 | Serpinb6c | 2.4456 | 4.7009 | 1.41E-07 | 6.11E-10 |
| 1980092 | NM_146135.1 | Pias3 | 1.0933 | 1.3314 | 1.52E-07 | 2.98E-08 |
| 4920427 | AK007145 | 1700108N18Rik | 0.9926 | 1.1499 | 1.80E-07 | 5.37E-08 |
| 1300475 | NM_009291.1 | Stra6 | 1.2628 | 2.6503 | 1.84E-07 | 3.84E-10 |
| 5390743 | NM_016959.2 | Rps3a | 1.3555 | 1.7995 | 2.84E-07 | 2.75E-08 |
| 7320685 | NM_008012.1 | Akr1b8 | 2.1012 | 3.0862 | 2.95E-07 | 1.23E-08 |
| 2340575 | NM_022983.2 | Edg7 | 1.3994 | 2.2987 | 4.08E-07 | 6.76E-09 |
| 6510309 | NM_001003963.1 | Dnmt3b | 0.6842 | 1.0312 | 4.98E-07 | 1.70E-08 |
| 610315 | NM_177630.2 | BC058638 | 1.0912 | 1.6642 | 5.08E-07 | 1.57E-08 |
| 870309 | AK045954 | Serpine2 | 1.7191 | 2.9890 | 5.12E-07 | 5.31E-09 |
| 6370047 | NM_175657.1 | Hist1h4m | 1.1178 | 1.7027 | 6.46E-07 | 2.03E-08 |
| 4490445 | NM_010474.1 | Hs3st1 | 1.5447 | 3.9086 | 6.87E-07 | 3.09E-10 |
| 1450066 | NM_025384.3 | Dnajc15 | 1.5744 | 2.767 | 7.03E-07 | 6.71E-09 |
| 2360519 | NM_013690.1 | Tek | 1.4452 | 3.244 | 1.01E-06 | 1.27E-09 |
| 3130079 | NM_177381 | Cog3 | 0.9544 | 1.5130 | 1.11E-06 | 2.53E-08 |
| 240025 | NM_177193.4 | Islr2 | 1.0036 | 1.5785 | 1.22E-06 | 2.99E-08 |
| 3710544 | NM_025569.1 | Mgst3 | 1.0799 | 2.0772 | 1.40E-06 | 6.46E-09 |
| 1300082 | XM_147240.1 | Clcn2 | 1.0485 | 1.9787 | 1.69E-06 | 9.17E-09 |

Yellow: BP=Developmental Process GO: 0032502

Red: BP= Development and Heart Development GO: 0007507

Silver: BP=Calcium Homeostatsis GO: 0055074 or ion transport GO: 0006811
